# A linguistic hesitant fuzzy group decision-making method for sustainable human-robot collaboration

**DOI:** 10.1371/journal.pone.0333758

**Published:** 2025-10-09

**Authors:** Xuejiao Zhang, Yu Yang, Qian Chen, Jing Wang

**Affiliations:** 1 College of Mechanical and Vehicle Engineering, Chongqing University, Chongqing, China; 2 School of Economics and Management, Tsinghua University, Beijing, China; 3 Research Center of Technological Innovation, Tsinghua University, Beijing, China; Gonbad Kavous University, IRAN, ISLAMIC REPUBLIC OF

## Abstract

In intelligent manufacturing for complex products, the configuration and allocation of human-robot collaboration units (HRCUs) are of critical importance for enhancing production performance. To address the insufficient research on the impact of individual irrational behaviors and group-reference behaviors in HRCUs construction, a stable one-to-many human-robot-position matching decision-making (HRPMDM) method in hesitant fuzzy environments is proposed. Specifically, linguistic hesitant fuzzy sets (LHFSs) are adopted to characterize evaluators’ dual uncertainties in linguistic term selection and membership degree assignment. Subsequently, the Cloud Model is adopted to quantitatively transform the LHFSs, thereby providing support for the proposed clustering algorithm based on cognitive similarity, enabling it to divide the matching objects into several subgroups according to the degree of cognitive similarity among individuals. Furthermore, to reduce the bias in attribute weight assessment caused by peer effects, a social network-based $DIL-W^{\alpha }$ algorithm that enables precise quantification of subgroup and member weights is proposed. These quantified weights are then integrated into the group consensus adjustment process to provide reliable reference correction values for individual assessments. Additionally, multi-proposition belief structures are introduced to represent uncertain matching preference rankings (UMPRs) influenced by group reference behaviors, and a corresponding satisfaction measurement method is further developed. Finally, a practical case study demonstrates the operational feasibility and performance efficacy of the proposed method. This study is the first to integrate carbon neutrality cost optimization objectives into human-robot matching decisions and develops a Quality of Service (QoS)-optimized allocation strategy for HRCUs in heterogeneous production environments. The results demonstrate that the proposed matching method has led to significant improvements in both production efficiency and environmental sustainability for complex product manufacturing.

## 1. Introduction

As a core application domain of bilateral matching theory, the person-position matching framework was originally established by Caplan [1]. Subsequent research has extended this theory by constructing a bilateral matching decision-making (BMDM) model based on utility maximization and behavioral theory [2]. The enhanced model incorporates several advanced elements, such as hesitant fuzzy evaluation [3,4], multi-reference point gain-loss assessment [5], and satisfaction function with time attenuation characteristics [6], thereby providing quantitative theoretical support for production resource allocation decisions. In the Industry 4.0 transformation, industrial robots equipped with diverse tools and sensors have been widely deployed across manufacturing operations [7,8]. To establish an industrial workspace where humans and robots coexist, the concept of human-robot collaboration has been introduced and gained significant attention in industrial sector [9]. This technological advancement has transformed traditional person-position matching decision-making (PPMDM) into HRPMDM. Based on the level of physical interaction, human-robot collaboration can be categorized into two primary modes [10]. In indirect collaboration, humans and robots operate as independent units that cooperate by handling distinct subtasks or alternately performing identical subtasks, while in direct collaboration, they serve as complementary production elements that jointly participate in every phase of the task execution.

In conventional research, robots typically replace humans in performing high-intensity and high-risk tasks [11,12], e.g., welding, material handling, where humans and robots are treated as competitive operational units [13]. However, in manufacturing complex products with unstructured tasks, industrial robots exhibit inherent technical limitations, particularly in anomaly handling, which severely constrain production efficiency [14]. To mitigate these limitations, industry has increasingly adopted hybrid automated production line that combine human cognitive flexibility with robotic operational precision, thereby enabling the sharing of workspace and tasks [15]. Nevertheless, current research on HRPMDM focuses only on the first mode [16]. Therefore, to address the requirements of human-robot collaborative operations in complex production lines, it is important to establish a matching method between HRCUs and positions under shared tasks and workspace constraints, which has significant theoretical and practical value for the development of intelligent manufacturing.

However, it should be noted that (1) **existing studies have primarily focused on numerical hesitation in membership degree representation, while neglecting the semantic hesitation characteristics exhibited by matching entities during linguistic term selection.** When evaluating subjects make hesitant fuzzy assessments of evaluation criteria, their judgments based on fragmented information often exhibit significant uncertainty due to cognitive limitations and information incompleteness. To overcome the limitations of traditional precise numerical methods in characterizing hesitation [17], researchers proposed hesitant fuzzy set (HFS) theory, which has subsequently developed various extended models [18,19]. Notably, for decision-makers demonstrating hesitation in linguistic term selection, the hesitant fuzzy linguistic term set (HFLTS) provides a more accurate representation of semantic-level uncertainty [20]. However, the model’s requiring the sum of membership degrees for all linguistic terms to equal 1 has proven difficult to implement for decision-makers with limited cognitive capacity and practical experience. (2) **In the decision-making process of human-robot-position matching, the weights of evaluation criteria play a crucial role. Existing studies have largely overlooked the impact of human irrational behavior on attribute weight determination.** In reality, organizational members form complex social networks through multi-channel interactions, where cognitively similar individuals spontaneously cluster into subgroups [21,22]. Under peer effects, subgroup members tend to adopt peers’ viewpoints [23]. When majority consensus emerges, this agreement exerts conformity pressure on dissenters, compelling expressions incongruent with their true positions. Research indicates such irrational behaviors intensify group information homogenization, ultimately inducing matching decision biases. (3) **In calculating matching utility values, existing studies have primarily focused on individuals’ gain-loss analysis based on expectation reference points, while neglecting the psychological perception factors toward potential matching objects.** Failing to match with superior alternatives induces disappointment, whereas avoiding inferior matches elicits relief [2,24]. Consequently, incorporating disappointment and elation values derived from psychological perceptions into utility calculations becomes essential for enhancing matching effectiveness. (4) **Existing research has neglected the impact of group-reference psychological behaviors on individuals, resulting in uncertainty in matching preference rankings (MPRs).** As a key criterion for evaluating matching satisfaction, existing studies primarily determine MPRs based on utility values. However, when utility differences between matching alternatives fall below a certain threshold, individuals struggle to accurately reflect true preferences through strong preference orders. Moreover, individuals may adjust initial preference rankings by referencing subgroup members’ preferences. Currently, effective methods for computing matching satisfaction based on weak preference orders remain lacking. (5) **The widespread application of human-robot collaboration units (HRCUs) has been demonstrated to effectively mitigate environmental pollution** [25]**. However, current research has not sufficiently explored how to reduce carbon neutrality costs through optimized HRCUs configuration.** In sustainable economic environment, enterprises are actively adopting carbon emission offset and removal technologies to achieve net-zero targets [26,27]. Notably, when employees’ operational skills and knowledge fail to maintain robots in optimal operating conditions, accelerated equipment wear may occur, subsequently increasing energy consumption and carbon emissions.

Based on the above deficiencies, the research motivations of this paper are as follows:

(1) Due to cognitive and experiential limitations, individuals encounter difficulties in accurately selecting linguistic terms and determining membership degrees. To address this, HFLTs are employed for evaluating indicator values by integrating multiple linguistic terms and reflecting their possible membership degree distributions, thereby overcoming the constraints of traditional methods in expressing individual matching preferences. Furthermore, a cloud model is introduced to compute distances between HFLTSs with varying numbers of linguistic terms, mitigating information distortion during transmission.
(2) For decision-makers exhibiting both opinion convergence tendencies and susceptibility to group pressure, it is essential to propose a social network analysis-based group consensus feedback adjustment method. The approach provides quantitative criteria for modifying individual evaluation information, effectively mitigating indicator weight bias caused by group information homogenization.
(3) Utility value is an important factor for determining an individual’s MPRs. Therefore, it is necessary to incorporate both anticipated disappointment and elation emotions from potential matches into the utility function calculation, so as to achieve a precise measurement of bilateral matching intentions.
(4) Considering the group reference behavior of individuals, a novel approach is required to assess MPRs when it is partially or entirely unknown. This addresses the challenge of accurately ranking similar alternatives within the individual decision-making process.
(5) In continuous manufacturing environments, carbon neutrality costs are primarily determined by unit carbon dioxide removal costs and carbon emission volumes [28]. Therefore, when constructing HRCUs, both the workers’ and robots’ unit carbon dioxide removal costs and emission levels have to be incorporated to minimize the carbon neutrality costs generated by potential HRCUs.

The purpose of this study is to construct a novel HRPMDM method for fuzzy-dual hesitant environments, addressing the construction challenges of HRCUs in sustainable complex product manufacturing, with the aim of achieving efficient and low-carbon production matching. The proposed method analyzes the impacts of peer effects and group reference behaviors on individuals to construct a one-to-many stable matching model with dual optimization objectives: maximizing matching satisfaction and minimizing carbon neutrality costs. Based on the model solutions, optimal HRCUs are allocated to workstations with diverse structural configurations, thereby effectively ensuring the fulfillment of QoS requirements.

The novelty of this study is manifested through two dimensions: problem innovation and methodological innovation. (1) A HRCUs matching model is constructed for fuzzy-dual hesitant environments, providing new perspectives for carbon neutrality cost reduction and production task allocation. (2) In the multi-attribute decision-making (MADM) process, by analyzing individual irrational behavior and group-reference behavior, we propose a group consensus feedback-based attribute weighting method and an UMPRs-based satisfaction evaluation method, which not only offers practical guidance for efficient production of complex products in sustainable manufacturing environments, but also advances group decision-making (GDM) theory in complex scenarios.

The remainder of this paper is organized as follows. Section 2 establishes the fundamental concepts and methodological foundations for the matching problem. Section 3 elaborates on the construction of HRCUs and their configuration schemes for workstations with diverse structural layouts. Section 4 validates the proposed model’s feasibility and practical value through a case study of a new energy vehicle hybrid automation production line. Section 5 demonstrates the advantages and effectiveness of the proposed method via numerical simulations and comparative analyses. Finally, Section 6 summarizes the study’s main contributions, limitations, and future research directions.

## 2. Preliminaries

In this section, the fundamental concepts and terminology that will be used are introduced. These definitions and notations form the foundation for understanding the subsequent sections.

### 2.1. Description of one-to-many bilateral matching

Traditional bilateral matching research is limited to one-to-one matching relationships. To characterize scenarios where an agent on one side needs to match with multiple agents on the opposite side, the concept of one-to-many bilateral matching is provided. In addition, the notion of blocking pairs is introduced to identify and eliminate unstable matching pairs, thereby ensuring the rationality of matching outcomes.

**Definition 1** [29]: Let $S=\{S_{i}|i,g=1,2,\cdots m,i\neq g\}$ and $T=\{T_{j}|j,l=1,2,\cdots n,j\neq l\}$ be two mutually exclusive sets of matching objects. A one-to-many bilateral matching between S and T is a mapping μ:S∪T→S∪T, satisfying the following conditions for all $S_{i}\in S$ and $T_{j}\in T$: (1) $\left|\mu (T_{j})=1\right|$, $\left|\mu (S_{i})\leq n\right|$; (2) $\mu (T_{j})=S_{i}$, $\mu (S_{i})=T\cup \{S_{i}\}$; (3) $\mu (S_{i})\in T_{j}$, $\mu (T_{j})=S_{i}$ and $\mu (S_{i})\cap \mu (S_{g})=\emptyset$. Specially, $\mu (T_{j})=S_{i}$ means $T_{j}$ is matched with $S_{i}$, and $\mu (S_{i})\in T_{j}$ means $T_{j}$ is one of objects that matched with $S_{i}$. $\left|\mu (T_{j})\right|$ is the number of objects that match with $T_{j}$. Likewise, $\left|\mu (S_{i})\right|$ is the number of $T_{j}$ that match with $S_{i}$.

**Definition 2** [30]: In a one-to-many bilateral matching between two objects, a blocking pair $\left(T_{j},S_{i}\right)$ exists in the matching mapping μ if either of the following conditions holds: (1) there exist $T_{j},T_{l}\in T$ and $S_{i},S_{g}\in S$ with $\mu (T_{j})=S_{i}$ and $\mu (T_{l})\in S_{g}$ such that $\alpha_{ji}^{T}\leq \alpha_{jg}^{T}$ and $\beta_{ji}^{S}\leq \beta_{li}^{S}$; (2) there exist $T_{j},T_{l}\in T$ and $S_{i},S_{g}\in S$ with $\mu (T_{j})=S_{i}$ and $\mu (S_{g})=S_{g}$ such that $\alpha_{ji}^{T}\leq \alpha_{jg}^{T}$. Specially, $\alpha_{ji}^{T}$ and $\beta_{ji}^{S}$ respectively represent the satisfaction degree of one matching object toward the other.

### 2.2. Conception of the cloud model

The conception of Cloud Model was first appeared in [13] to express the hesitancy and ambiguity that exist in natural language term set by its numerical characteristic of three levels.

**Definition 3** [31]: Let U be the universe of discourse, and T is a qualitative concept in U. For a random variable x belonging to T, where x∈X and X∈U, if x follows $x\sim N(E_{x},{E_{n}^{\prime }}^{2})$ and $E_{n}^{\prime }\sim N(E_{n},{H_{e}}^{2})$, then the certainty degree y(y∈[0,1]) of x∈T is defined as $y=e^{\frac{{\left(x-Ex\right)}^{2}}{2{\left({E^{\prime }}_{n}\right)}^{2}}}$. The distribution of x is called a normal Cloud, denoted by $C=(E_{x},E_{n},He)$, where each (x,y) pair constitutes a Cloud drop. The features of qualitative concept T can be described by the expectation Ex, entropy $E_{n}$, and hyper entropy $H_{e}$ in C. To calculate the fuzzy distance between two Clouds, an estimated score is provided for of each Cloud Model.

**Definition 4** [32]: Let s=xy represent the contribution of Cloud drops (x,y) to the qualitative conception. The estimated score of $\beta_{\xi }$ identified by a Monte Carlo simulation is shown as follows:


$$\hat{S}(\beta_{\xi })=\frac{\text{1}}{n}\sum_{\xi =1}^{n}x_{\xi }(exp(-\frac{\left(x_{\xi }-{E_{x}}_{\xi }\right)}{2{\left(E{n^{\prime }}_{\xi }\right)}^{2}}))$$ (1)

**Definition 5** [33]: Based on the fuzzy distance between two Clouds, the similarity degree of $\beta_{\xi }$ and $\beta_{\xi +1}$ is obtained by:


$$D(\beta_{\xi },\beta_{\xi +1})=(\left|E_{x_{\xi }}-E_{x_{\xi +1}}\right|,\left|E_{n_{\xi }}-E_{n_{\xi +1}}\right|,\left|E_{e_{\xi }}-E_{e_{\xi +1}}\right|)$$ (2)

$$\sin(\beta_{\xi },\beta_{\xi +1})=1-\frac{\hat{S}(D(\beta_{\xi },\beta_{\xi +1}))}{\hat{S}(\beta_{\xi })+\hat{S}(\beta_{\xi +1})}$$ (3)

**Definition 6** [34–36]: Three kinds of averaging operators: the Cloud weighted averaging (CWA), the Cloud ordered weighted averaging (COWA) and the Cloud hybrid averaging (CHA) for Clouds $\beta_{\xi }=(E_{x_{\xi }},E_{n_{\xi }},He_{\xi })(\xi =1,2,\cdots n)$ with the weight vector of Cloud $\omega_{\xi }\in \left[0,1\right]$ are shown as follows:


$$CWA_{\omega }(\beta_{1},\beta_{2},\cdots ,\beta_{n})=\sum_{\xi =1}^{n}\omega_{\xi }\beta_{\xi }=\left(\sum_{\xi =1}^{n}\omega_{\xi }E_{x_{\xi }},\sqrt{\sum_{i=1}^{n}\omega_{\xi }{E_{n_{\xi }}}^{2}},\sqrt{\sum_{i=\xi }^{n}\omega_{\xi }H{e_{\xi }}^{2}}\right)$$ (4)

$$COWA_{w}(\beta_{1},\beta_{2},\cdots ,\beta_{n})=\sum_{\xi =1}^{n}w_{\xi }{\dot{\beta }}_{\rho {(}_{\xi })}=\left(\sum_{\xi =1}^{n}w_{\xi }{\dot{E}}_{x_{\rho {(}_{\xi })}},\sqrt{\sum_{\xi =1}^{n}w_{\xi }{\dot{E}}_{n_{\rho {(}_{\xi })}}},\sqrt{\sum_{\xi =1}^{n}w_{\xi }\dot{H}{e_{\rho {(}_{\xi })}}^{2}}\right)$$ (5)

$$CHA_{w,\omega }(\beta_{1},\beta_{2},\cdots ,\beta_{n})=\sum_{\xi =1}^{n}w_{\xi }{\ddot{\beta }}_{\rho {(}_{\xi })}=\left(\sum_{\xi =1}^{n}w_{\xi }{\ddot{E}}_{x_{\rho {(}_{\xi })}},\sqrt{\sum_{\xi =1}^{n}w_{\xi }{\ddot{E}}_{n_{\rho {(}_{\xi })}}},\sqrt{\sum_{\xi =1}^{n}w_{\xi }\ddot{H}{e_{\rho {(}_{\xi })}}^{2}}\right)$$ (6)

### 2.3. Disappointment theory

Disappointment Theory was first proposed in [37], demonstrating that individuals compare the actual value obtained from their current matching choice with the expected value of alternative options. When the ratio of actual-to-expected value is less than 1, the matching objects feel regretful to make the current decision, otherwise, they feel pleased. Since then, other scholars have further extended the theory [38]. In general, decision makers are more sensitive to regret, which is called disappointment aversion [39]. Owing to the good performance in explaining and describing individuals’ psychological feelings, Disappointment Theory has become a predominant framework in behavioral decision research.

**Definition 7** [40]: Let the matching objects be ranked by the order of decreasing preference $S_{1}\geq S_{2}\geq \cdots \geq S_{n}$. The modified utility $U(\chi_{T})$ composed by the disappointment function $D(\chi_{T})$ and satisfies function $E(\chi_{T})$ is calculated as follows:


$$U(\chi_{T})=V(\chi_{T})-\left(\sum_{\tau =1}^{n}p_{\tau }D(V(\chi_{T})-V(\chi_{T}))\right)+\left(\sum_{\tau =1}^{n}p_{\tau }E(V(\chi_{T})-V(\chi_{T}))\right)$$ (7)

## 3. Proposed approach

### 3.1. The problem description and resolution framework

**Fig 1:**
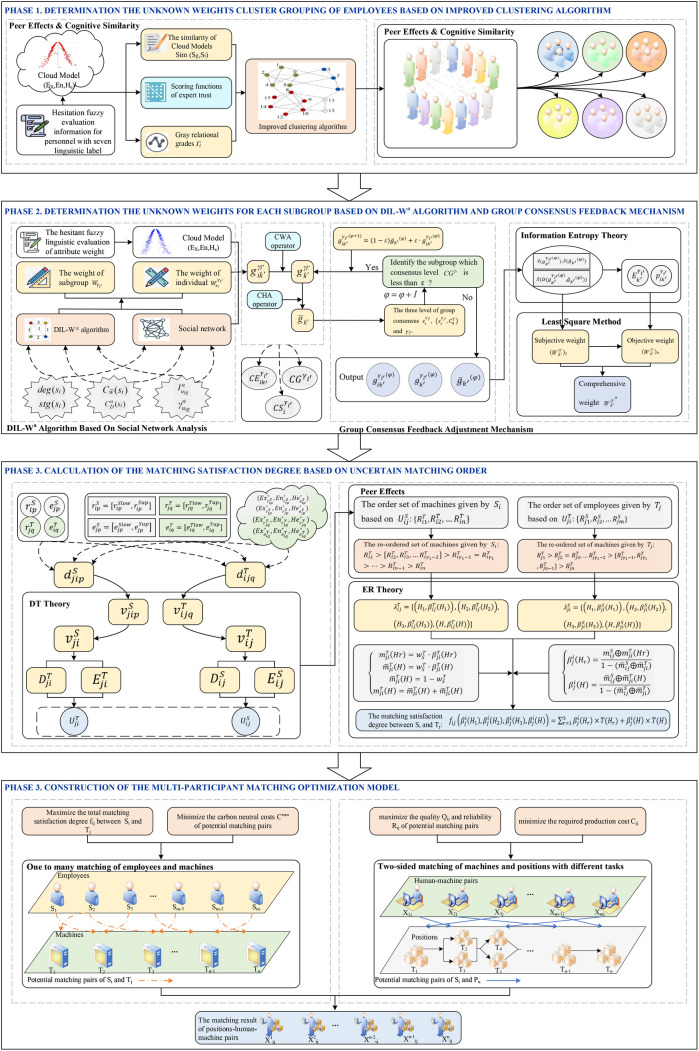
Proposed research framework for constructing and allocating HRCUs.

**Fig 2:**
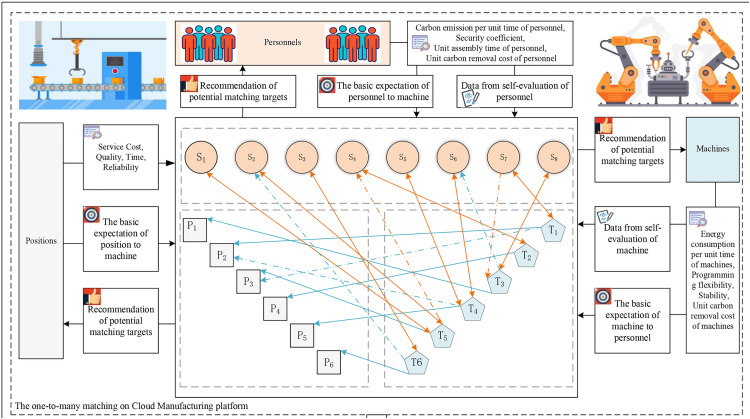
Process of HRCUs construction and position allocation in intelligent production lines.

The matching system comprises three distinct matching object sets: the employee set $S=\{S_{i}|i,g=1,2,\cdots ,m,i\neq g\}$, $S_{i}$ represents the i−th employee distinct from all $S_{g}$, the robot set $T=\{T_{j}|j,l=1,2,\cdots ,m,i\neq l\}$ $T_{j}$ being the j−th robot $T_{l}$$U=\{U_{r}|r,t=1,2,\cdots ,z,r\neq t\}$ comprising unique positions $U_{r}\neq U_{t}$. Let m and n denote the number of employees and robots, respectively. These resources are allocated to z positions in an intelligent production line, where m,n≥2 and z≥m≥n. In this paper, MPRs are established between employees and robots. Positions with heterogeneous tasks generate independent preferences for HRCUs. The research framework is presented in Fig 1, and the process of HRCUs formation and position allocation is shown in Fig 2. Additionally, to formulate the proposed problem, the symbols used in the subsequent approach are described in Table A.1 (see S1 File).

### 3.2. Object clustering using cloud model similarity

In human-involved matching decision-making, individuals do not exist in isolation but rather interact as special resources endowed with social attributes. Such interactions frequently lead to the emergence of group clustering phenomena. Social cognitive theory demonstrates that individuals naturally tend to form clusters with others sharing similar cognitive structures during decision-making processes. Neglecting this clustering effect would reduce the accuracy of matching results. This study adopts individual’s cognitive levels toward low-carbon production as the grouping criterion, with particular focus on the impact of clustering phenomena on attribute weight assessment and matching prioritization. Furthermore, in hesitant fuzzy environments, human expressions typically utilize multiple linguistic terms assigned varying degrees of importance. This characteristic can be effectively represented by LHFSs.

Let $G=\{g_{0},g_{1},\cdots ,g_{v}\}$ be a linguistic term set. An LHFSs is denoted as: $L^{G}=\{\left(g_{v},lh(g_{v})\right)|g_{v}\in G\}$, where $lh(g_{v})=\{p_{m}|p_{m}\in [0,1]\}$ represents the set of related membership degrees used to quantify the hesitation degree of decision-maker when evaluating different linguistic variables [41]. On this basis, the LHFSs can be converted into the corresponding Cloud models as follows:


$$Ex_{L^{G}}^{*}=\frac{\text{1}}{\left|index(L^{G})\right|}\left(\sum_{v\in index(L^{G})}\frac{Ex_{v}}{lh(g_{v})}(\sum_{p\in lh(g_{v})}p)\right)$$ (8)

$$En_{L^{G}}^{*}=\sqrt{\frac{\text{1}}{\left|index(L^{G})\right|}\left(\sum_{v\in index(L^{G})}{\left(En_{v}\right)}^{2}\right)}$$ (9)

$$He_{L^{G}}^{*}=\sqrt{\frac{\text{1}}{\left|index(L^{G})\right|}\left(\sum_{v\in index(L^{G})}{\left(He_{v}\right)}^{2}\right)}$$ (10)

For example, the LHFSs of $\{(b_{1},0.2),(b_{3},0.7\}$ and $\{(b_{4},0.4),(b_{6},0.6,0.7\}$ can be transformed into the Cloud Model (1.25,0.93,0.44) and (2.32,1.03,0.36), which are in the given universe [0,5].

**Fig 3:**
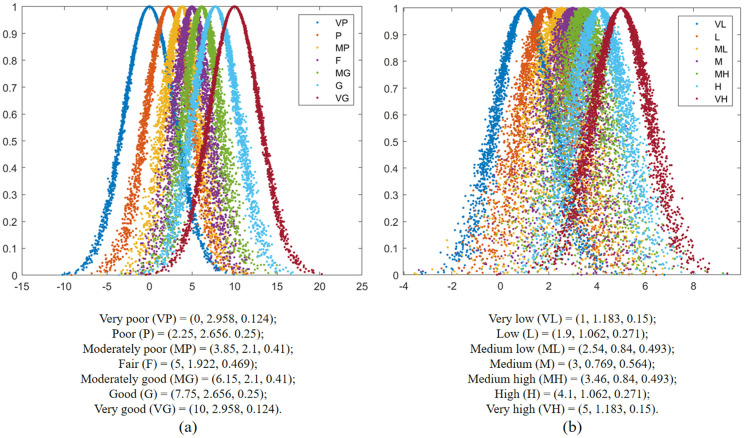
Cloud Model representation of seven-label linguistic term set. (a): Seven-label linguistic term set with $X_{\min}=0$, $X_{\max}=5$. (b): Seven-label linguistic term set with $X_{\min}=0$, $X_{\max}=10$.

Generally, a seven-label term set $G=\{g_{i}|i=-3,\cdots 0,\cdots 3,h\in N^{*}\}$ is used to represent the potential values of a linguistic variable. Let $f:g_{i}\to \theta_{i}$ denote the function of a linguistic scale that satisfies the conditions: (1) ifj<i, the set is ordered such that $g_{j}<g_{i}$; (2) The negation operator is defined as $g_{j}<neg(g_{i})=g_{-i}$. The Cloud Models for a seven-label linguistic term set with different values of $X_{\max}$ and $X_{\min}$ are illustrated in Fig 3.

Based on this, a gray relational analysis method based on Cloud Model similarity is proposed to accurately group decision-makers. The gray relational coefficient between $S_{i}$ and $S_{g}$ is obtained by


$$\alpha (i,k)=\frac{\underset{i}{\min}\underset{k}{\min}\Delta gi+\rho \underset{i}{\max}\underset{k}{\max}\Delta gi}{\Delta gi+\rho \underset{i}{\max}\underset{k}{\max}\Delta gi}\;\;i,g=1,2,\cdots m,i\neq g,k=1,2,\cdots l$$ (11)

where $\Delta gi=\left|1-sim(S_{g},S_{i})\right|$. Typically, the distinguishable coefficient ρ is set to 0.5, where ρ∈[0,1].

Then, gray relational grade between $S_{i}$ and reference sequence is calculated as:


$$\Gamma_{i}=\sum_{k=1}^{l}\sum_{i=1}^{m}w_{k}^{E}\alpha (i,k)$$ (12)

where the expert weights $w_{k}^{E}$ are derived from the scoring functions proposed in [32]. The intuitive trust information matrix among experts, denoted as $V=(V_{kf}{)}_{b\times l}$ is provided in Table A.4 (see S1 File). Finally, $S_{i}$ is clustered using the clustering algorithm described in the pseudocode below.

**Input:** The similar degree matrix between $S_{i}$ and $S_{g}$, the threshold for clustering φ.

**Output:** The number of groups L, the number of $S_{i}$ in each group $N_{1},N_{2},\cdots N_{L}$.

1. Allocate each gray relational grade matrix of $S_{i}$ to its own cluster $T_{k},k=1,2,\cdots ,p$. Define L=m and $N_{k}=1$ for all k∈{1,2,⋯,L}.

2. Compute an upper triangular matrix $(S_{i,g}{)}_{L\times L}$, where i<g. $S_{i,g}$ is gray relational grade between two cluster $T_{i}$ and $T_{g}$.

3. Find the biggest pair of clusters $S_{\max}(i^{\prime },g^{\prime })=\max_{1\leq i^{\prime },g^{\prime }\leq G}(S_{i^{\prime },g^{\prime }})$.

4. **Where** $S_{\max}(i^{\prime },g^{\prime })\geq \delta$ **do**

  a) Merge Cluster $T_{i}$ and $T_{g}$ into a new group.

  b) **If** $l=i^{\prime }$ **then**

     $N_{l}=N_{i^{\prime }}+N_{g^{\prime }}$

     **else if** $g^{\prime }\leq l\leq L-1$ **then**

      $N_{l}=N_{l+1}$

     **end if**

  c) L←L−1

  d) Compute upper triangular matrix $(S_{i,g}{)}_{L\times L}$ of the remaining clusters.

  e) Find the biggest pair of clusters $S_{\max}(i^{\prime },g^{\prime })=\max_{1\leq i^{\prime },g^{\prime }\leq G}(S_{i^{\prime },g^{\prime }})$

  f) **If** no clusters left to merge **then**

     **break**

     **end if**

**end while**

**return** $L,N_{1},N_{2},\cdots ,N_{L}$.

### 3.3. Attribute weight determination considering individual irrationality

In matching decision-making processes, the determination of attribute weights plays a pivotal role in influencing the rationality and effectiveness of final decisions. Traditional decision-making models typically assume that attribute weights are predetermined and known in advance [42,43]. However, this assumption often proves unrealistic in practice. In matching decision processes, clustering effects lead to the natural formation of groups comprising individuals with similar cognitive patterns. Consequently, it is necessary to obtain the evaluation values of attribute weights from different subgroups. Nevertheless, different groups assess attribute weights differently due to their unique cognitive perspectives. These variations also reflect irrational biases caused by social influences like peer effects. To mitigate extreme evaluation biases caused by irrational behaviors, this section proposes a consensus-based dynamic adjustment mechanism for attribute weight determination. This mechanism performs calibrated modifications to initial weight assessments from different subgroups, thereby significantly enhancing the accuracy of weight evaluation.

#### 3.3.1. Social network-based subgroup weight determination.

To accurately compute the group consensus evaluation based on subgroup assessments, it is essential to determine the relative importance of each subgroup, thereby establishing their respective weightings in the consensus calculation. To overcome the limitation of determining subgroup weights solely based on group size, an $DIL-W^{\alpha }$ algorithm is developed to determine the weights of subgroups by analyzing the strength of connections between individuals within the social network.

We assume the social network among members is an undirected and weighted network represented as F=(S,R,U), where $\left\{S_{i}|i=1,2,\cdots ,m\right\}$ is the set of nodes, and $R=\{r_{ig}|i,g=1,2,\cdots ,m,i\neq g\}$ is the set of undirected edges. The edge weights are defined by the set $U=\{u_{ig}|i,g=1,2,\cdots m,i\neq g\}$, where each weight $u_{ig}$ quantifies the social relationship strength between nodes $S_{i}$ and $S_{g}$. Based on the node importance measures, we calculate the weight for each $S_{i}$ using an improved $DIL-W^{\alpha }$ algorithm. For centrality comparison between nodes [44], our algorithm considers the number of nodes connected to the target node, differing from the approach in [45]. The degree of node $S_{i}$, denoted as $deg(S_{i})$, is determined by counting its connected nodes. Similarly, the strength of node $S_{i}$, represented as $stg(S_{i})$, is calculated as the sum of the weights for all edges incident to node $S_{i}$. Formally, this is expressed as:


$$stg(S_{i})=\sum_{s_{g}\in N(S_{i})}u_{ig}$$ (13)

The average weight of node $S_{i}$ is defined as:


$$C_{\bar{W}}^{\alpha }(S_{i})=(\frac{\sum_{S_{g}\in N(S_{i})}u_{ig}}{deg(S_{i})}{)}^{\alpha }\;i,g=1,2,\cdots ,m$$ (14)

where α is a tuning parameter for adjustment.

Let $C_{D}^{\alpha }(S_{i})$ denote the centrality of node $S_{i}$ in the weighted network, which is computed by:


$$C_{D}^{\alpha }(S_{i})=deg(S_{i})\cdot C_{\bar{w}}^{\alpha }(S_{i})$$ (15)

The tuning parameter α balances the relationship between node degree and strength. When α=0, centrality depends solely on the degree $deg(S_{i})$. When α=1, centrality is determined exclusively by edge strength $\sum_{s_{g}\in N(S_{i})}u_{ig}$.

The importance of edge $r_{ig}$ between node $S_{i}$ and $S_{g}$ is calculated as follows:


$$I_{u_{ig}}^{\alpha }=\frac{2\cdot (C_{D}^{\alpha }(s_{i})-b_{i}^{\alpha })\cdot (C_{D}^{\alpha }(s_{g})-b_{g}^{\alpha })}{p^{\left(1-\alpha \right)}{\left(u_{i}+u_{g}-2u_{ig}\right)}^{\alpha }+2}$$ (16)

where $b_{h}^{\alpha }=p^{\left(1+\alpha \right)}\cdot u_{h}^{\alpha }$ with h∈{i,g}. Here, p represents the number of triangles containing edge $r_{ig}$, and $u_{k}$ denotes the total weight of the edges connected to node $S_{k}$ that form triangles with edge $r_{ig}$. The contribution of node $S_{k}\in S$ to the importance of edge $r_{ig}$ is calculated as follows:


$$\gamma_{u_{ig}}^{\alpha }=I_{u_{ig}}^{\alpha }\cdot \frac{C_{D}^{\alpha }(s_{i})-C_{\bar{W}}^{\alpha }(s_{i})}{\left(C_{D}^{\alpha }(s_{i})-C_{D}^{\alpha }(s_{g}))-(C_{\bar{W}}^{\alpha }(s_{g})-C_{\bar{W}}^{\alpha }(s_{i})\right)}$$ (17)

The importance of node $S_{i}$ is represented as follows:


$$\tau_{S_{i}}^{\alpha }=C_{D}^{\alpha }(S_{i})+\sum_{s_{g}\in N(S_{i})}\gamma_{u_{ig}}^{\alpha }$$ (18)

Furthermore, we determine the weight of each subgroup member $w_{S_{i}}^{\gamma_{l^{\prime }}}$ through social network analysis which is presented as follows:


$$w_{S_{i}}^{\gamma_{l^{\prime }}}=\frac{\tau_{S_{i}}^{\alpha }}{\sum {\mathrm{\backslash nolimits}}_{i=1}^{m}\tau_{S_{i}}^{\alpha }}$$ (19)

Finally, the subgroup weight $w_{\gamma_{l^{\prime }}}$ is computed as follows:


$$w_{\gamma_{l^{\prime }}}=\sum_{i=1}^{nl}w_{S_{i}}^{\gamma_{l^{\prime }}}\;l^{\prime }=1,2,\cdots ,L$$ (20)

The above formula demonstrates that for subgroups with equal numbers of $S_{i}$, their weights scale proportionally to their constituent $S_{i}$ weights. This design intentionally prevents smaller subgroups with fewer $S_{i}$ from being unduly dominated by larger ones.

#### 3.3.2. Group consensus-based attribute weight determination.

Considering that individual evaluations of different indicators may be influenced by peers within the group, the direct weighting of individual criterion assessments may lead to inaccurate attribute weight evaluations. Therefore, a group consensus feedback adjustment mechanism is proposed in this study to determine the weights of multiple attributes. The evaluation attributes demonstrate that the importance of criteria $C^{T}$, as assessed by subgroup members, is represented by LHFSs. This linguistic representation can be further converted into a Cloud Model matrix as follows:


$$G_{ik}=\{{\left(g_{ik^{\prime }}^{\gamma_{l^{\prime }}}\right)}_{nl\times q}|l^{\prime }=1,2,\cdots ,L,i=1,2,\cdots ,nl,k^{\prime }=1,2,\cdots ,q\}$$ (21)

Then CHA operator is employed to compute the criteria evaluation matrix $G^{\gamma_{l^{\prime }}}=\{(g_{k^{\prime }}^{\gamma_{l^{\prime }}}{)}_{l\times q}\}$ for subgroup $\gamma_{l^{\prime }}$ with respect to criteria $C_{q}^{T}$. Subsequently, given the precomputed subgroup weights $w_{\gamma_{l^{\prime }}}$, the collective evaluation matrix $\bar{G}=({\bar{g}}_{k^{\prime }}{)}_{l\times q}$ is derived as follows:


$$g_{k^{\prime }}^{\gamma_{l^{\prime }}}=\sum_{i=1}^{nl}w_{s_{i}}^{\gamma_{l^{\prime }}}g_{ik^{\prime }}^{\gamma_{l^{\prime }}}$$ (22)

$${\tilde{g}}_{k^{\prime }}^{\gamma_{l^{\prime }}}=L\cdot w_{\gamma_{l^{\prime }}}\cdot g_{k^{\prime }}^{\gamma_{l^{\prime }}}$$ (23)

$${\bar{g}}_{k^{\prime }}=L\cdot w_{\gamma_{l^{\prime }}}\cdot {\tilde{g}}_{k^{\prime }}^{\gamma_{l^{\prime }}}$$ (24)

where $g_{k^{\prime }}^{\gamma_{l^{\prime }}}$ represents the i−th largest Cloud Model transformed from subgroup weight. The weight set $\left\{{\tilde{w}}_{\gamma_{l^{\prime }}}|{\tilde{w}}_{\gamma_{l^{\prime }}}\in [0,1]\right\}$ is derived from a monotonic increasing function $\dot{F}(\varphi )$, subject to the condition $\sum_{l^{\prime }=1}^{L^{\prime }}{\tilde{w}}_{\gamma_{l^{\prime }}}=1$.


$${\tilde{w}}_{\gamma_{l^{\prime }}}=\dot{F}(\frac{l^{\prime }}{n})-\dot{F}(\frac{l^{\prime }-1}{n})$$ (25)

$$\dot{F}(\varphi )=\{\begin{array}{cc} {}*20c0 & \varphi <\alpha \\ \frac{\varphi -\alpha }{\beta -\alpha } & \alpha \leq \varphi \leq \beta \\ 1 & \varphi \geq \beta \end{array}$$ (26)

Generally, assuming the parameters (α,β)=(0.3,0.8) [46], when L=3, we obtain the weight vector $\tilde{w}=(0.0666,0.6667,0.2667)$. Using this computational framework, the group’s comprehensive evaluation value for attribute weight assessment is derived. To maximize group consensus, it is necessary to compute the discrepancies between individual evaluations and the comprehensive group evaluation at three hierarchical levels: evaluation elements, evaluation schemes, and evaluation matrices [47]. Subsequently, based on both $g^{\gamma_{l^{\prime }}}$ and ${\bar{g}}_{k^{\prime }}$, the consensus level for element $\left(s_{ik^{\prime }}^{\gamma_{l^{\prime }}},C_{q}^{T}\right)$ is defined as follows:


$$CE_{ik^{\prime }}^{\gamma_{l^{\prime }}}=1-S(g_{ik^{\prime }}^{\gamma_{l^{\prime }}},{\bar{g}}_{k^{\prime }})$$ (27)

The consensus level for element $\left(s_{i}^{\gamma_{l^{\prime }}}\right)$ is defined as:


$$CS_{i}^{\gamma_{l^{\prime }}}=\frac{\text{1}}{q}\sum_{k^{\prime }=1}^{q}CE_{ik^{\prime }}^{\gamma_{l^{\prime }}}$$ (28)

The individual consensus level of subgroup $\gamma_{l^{\prime }}$ is calculated as:


$$CG^{\gamma_{l^{\prime }}}=\frac{\text{1}}{nl}\sum_{i=1}^{nl}CS_{i}^{\gamma_{l^{\prime }}}=\frac{\text{1}}{nlq}\sum_{k^{\prime }=1}^{q}\sum_{i=1}^{nl}\left(1-S(g_{ik^{\prime }}^{\gamma_{l^{\prime }}},\bar{g}k^{\prime })\right)=1-\frac{\text{1}}{nlq}\sum_{k^{\prime }=1}^{q}\sum_{i=1}^{nl}\left(S(g_{ik^{\prime }}^{\gamma_{l^{\prime }}},\bar{g}k^{\prime })\right)$$ (29)

$$g_{ik^{\prime }}^{\gamma_{l^{\prime }}(\varphi +1)}=(1-\varepsilon ){\bar{g}}_{k^{\prime }}^{\left(\varphi \right)}+\varepsilon \cdot g_{ik^{\prime }}^{\gamma_{l^{\prime }}(\varphi )}$$ (30)

**Fig 4:**
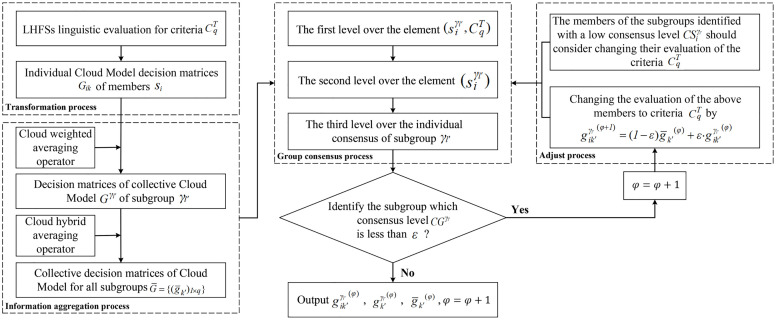
Group consensus feedback adjustment mechanism framework.

To bridge the discrepancy between individual and comprehensive evaluations, a predefined threshold ε is implemented to identify members with lower consensus levels. This threshold is typically set below 1, since complete consistency is unlikely to achieve. The threshold can be adjusted according to actual consistency requirements. In this study, we establish the consensus threshold at ε=0.8, designate the correction parameter as ε=0.5 and initialize the consensus correction time at φ=0. Though the group consensus feedback adjustment mechanism depicted in Fig 4, personalized adjustment recommendations are generated for the attribute weight evaluations submitted by individual participants.

#### 3.3.3. Integrated approach for subjective and objective attribute weighting.

After multiple rounds of iterations adjustment, we have derived the revised Cloud Model $w_{k^{\prime }}^{T}$ proposed by employee $S_{i}$ in subgroup $\gamma_{l^{\prime }}$. Subsequently, a new aggregated Cloud Model ${\bar{g}}_{k^{\prime }}^{\left(\varphi \right)}$ is obtained that meets the for group consensus degree threshold requirements. Finally, the objective weights of $C_{k^{\prime }}^{T}$ are calculated using the following optimal model:


$$\max Z=\sum_{k^{\prime }=1}^{q}{\left(w_{k^{\prime }}^{\gamma_{l^{\prime }}}\right)}_{s}\cdot S(g_{k^{\prime }}^{\gamma_{l^{\prime }}(\varphi )},{\bar{g}}_{k^{\prime }}^{\left(\varphi \right)})$$ (31)

$$S\left(g_{k^{\prime }}^{\gamma_{l^{\prime }}(\varphi )},\bar{g}{\left(\varphi \right)}_{k^{\prime }}\right)=1-\frac{\hat{s}(D(g_{k^{\prime }}^{\gamma_{l^{\prime }}(\varphi )},{g_{k^{\prime }}}^{\left(\varphi \right)}))}{\hat{s}(g_{k^{\prime }}^{\gamma_{l^{\prime }}(\varphi )})+\hat{s}({g_{k^{\prime }}}^{\left(\varphi \right)})}$$ (32)

$$\sum_{k^{\prime }=1}^{q}{\left(w_{k^{\prime }}^{\gamma_{l^{\prime }}}\right)}_{s}=1$$ (33)

$$\delta_{low}\leq (w_{k^{\prime }}^{\gamma_{l^{\prime }}}{)}_{s}\leq \delta_{high},\;(w_{k^{\prime }}^{\gamma_{l^{\prime }}}{)}_{s}\in \Omega$$ (34)

where Ω denotes the set of all five known weight forms. In this study, the weights are represented as intervals values: $\left\{0\leq \delta_{low}\leq w_{i}\right\}$ and $\left\{w_{i}\leq \delta_{high}\leq 1\right\}$, where $\delta_{high}=\delta_{high}+\varsigma =0.35$.

The objective weights are then derived from the subjective weights through information entropy theory, as expressed in the following equations:


$$p_{k^{\prime }}^{\gamma_{l^{\prime }}}=\frac{\hat{s}(D(g{ik^{\prime }\gamma_{l^{\prime }}}^{\left(\varphi \right)},{{\bar{g}}_{k^{\prime }}}^{\left(\varphi \right)}))}{\sum_{i=1}^{nl}\hat{s}(D(g{ik^{\prime }\gamma_{l^{\prime }}}^{\left(\varphi \right)},{{\bar{g}}_{k^{\prime }}}^{\left(\varphi \right)}))}$$ (35)

$$E_{k^{\prime }}^{\gamma_{l^{\prime }}}=-(\frac{\text{1}}{InL})\sum_{i=1}^{nl}p_{k^{\prime }}^{\gamma_{l^{\prime }}}Inp_{k^{\prime }}^{\gamma_{l^{\prime }}}$$ (36)

$$(w_{k^{\prime }}^{\gamma_{l^{\prime }}}{)}_{o}=\frac{1-E_{k^{\prime }}^{\gamma_{l^{\prime }}}}{q-\sum_{k^{\prime }=1}^{q}E_{k^{\prime }}^{\gamma_{l^{\prime }}}}$$ (37)

The optimal combination weights are obtained using the least-square method to achieve a balance of interests between both parties. To integrate two types of weights, the coefficients $a_{1}$ and $a_{2}$ can be determined by minimizing the dispersion of $w_{k^{\prime }}^{\gamma_{l^{\prime }}}$. The presented model is formulated as follows:


$$w_{k^{\prime }}^{\gamma_{l^{\prime }}}=a_{1}(w_{k^{\prime }}^{\gamma_{l^{\prime }}}{)}_{s}+a_{2}(w_{k^{\prime }}^{\gamma_{l^{\prime }}}{)}_{o}$$ (38)

$$\min Z=\left({\left\|w_{k^{\prime }}^{\gamma_{l^{\prime }}}-{\left(w_{k^{\prime }}^{\gamma_{l^{\prime }}}\right)}_{o}\right\|}_{2}\right)+\left({\left\|w_{k^{\prime }}^{\gamma_{l^{\prime }}}-{\left(w_{k^{\prime }}^{\gamma_{l^{\prime }}}\right)}_{s}\right\|}_{2}\right)$$ (39)

$$a_{1}(w_{k^{\prime }}^{\gamma_{l^{\prime }}}{)}_{o}(w_{k^{\prime }}^{\gamma_{l^{\prime }}}{)}_{s}^{T}+a_{2}(w_{k^{\prime }}^{\gamma_{l^{\prime }}}{)}_{o}(w_{k^{\prime }}^{\gamma_{l^{\prime }}}{)}_{o}^{T}=(w_{k^{\prime }}^{\gamma_{l^{\prime }}}{)}_{o}(w_{k^{\prime }}^{\gamma_{l^{\prime }}}{)}_{o}^{T}$$ (40)

$$a_{1}(w_{k^{\prime }}^{\gamma_{l^{\prime }}}{)}_{s}(w_{k^{\prime }}^{\gamma_{l^{\prime }}}{)}_{s}^{T}+a_{2}(w_{k^{\prime }}^{\gamma_{l^{\prime }}}{)}_{s}(w_{k^{\prime }}^{\gamma_{l^{\prime }}}{)}_{o}^{T}=(w_{k^{\prime }}^{\gamma_{l^{\prime }}}{)}_{s}(w_{k^{\prime }}^{\gamma_{l^{\prime }}}{)}_{s}^{T}$$ (41)

After obtaining the precise values of $a_{1}$ and $a_{2}$, they are normalized to derive the optimal comprehensive weight $w_{k^{\prime }}^{\gamma_{l^{\prime }}*}$.


$$w_{k^{\prime }}^{\gamma_{l^{\prime }}*}=\frac{a_{1}}{a_{1}+a_{2}}\cdot (w_{k^{\prime }}^{\gamma_{l^{\prime }}}{)}_{s}+\frac{a_{2}}{a_{1}+a_{2}}\cdot (w_{k^{\prime }}^{\gamma_{l^{\prime }}}{)}_{o}$$ (42)

### 3.4. Uncertain matching preference ranking evaluation and satisfaction measurement

Traditional research on bilateral matching typically determines matching satisfaction by comparing utility-based preference rankings. However, in actual decision-making processes, individuals exhibit not only psychological fluctuations arising from discrepancies between actual and expected outcomes, but also complex emotional responses, such as disappointment or elation triggered by comparative evaluations of potential matches’ utilities. More critically, reference-dependent behavior may lead decision-makers to revise their initial utility rankings, thereby introducing substantial preference uncertainty. To address these challenges, this section focuses on quantifying utility values under psychological perception effects and accurately computing matching satisfaction with UMPRs.

#### 3.4.1. Utility computation for Multi-format evaluations with psychological perception.

To characterize the heterogeneous features of evaluation attributes, three distinct representations: exact numbers, interval numbers, and Cloud Models derived from LHFSs are adopted. These representations capture both expected and actual values, enabling computation of perceived utility values for each potential matching pair. For consistency, all cost criteria are converted into benefit criteria, resulting in uniformly beneficial criteria. The criteria valves are categorized into three subsets based on their representation forms: crisp numbers $C^{R}$, interval numbers $C^{I}$, and linguistic labels $C^{L}$. Let $E^{S}=[e_{iq}^{S}{]}_{m\times g}$ be the employee expectation matrix for robot $C_{q}^{T}$, $R^{S}=[r_{ip}^{S}{]}_{m\times f}$ be the employee actual-value matrix for criteria $C_{p}^{S}$, $E^{T}=[e_{jp}^{T}{]}_{n\times f}$ be the robot expectation matrix for employee criteria $C_{p}^{S}$, and $R^{T}=[r_{jq}^{T}{]}_{n\times g}$ be robot actual-value matrix for criteria $C_{q}^{T}$.

Following the acquisition of matching object evaluation data, matching gains and losses are computed by measuring distances between corresponding expectation matrices $E^{T}$ and actual-value matrices $R^{T}$. The distance computation method differs according to criteria type. For crisp numbers $C_{f}^{S},C_{g}^{T}\in C^{R}$, $d_{jip}^{S}=\left|r_{ip}^{S}-e_{jp}^{S}\right|$ and $d_{ijq}^{T}=\left|r_{jq}^{T}-e_{iq}^{T}\right|$, where $d_{jip}^{S}$ quantifies the distance between employee $S_{i}$ ’s actual performance and robot $T_{j}$ ’s expected reference point for criteria $C_{p}^{S}$. For interval numbers $C_{f}^{S},C_{g}^{T}\in C^{I}$, given the interval representations: $r_{ip}^{S}=[r_{ip}^{S^{low}},r_{ip}^{S^{up}}]$, $e_{jp}^{S}=[e_{jp}^{S^{low}},e_{jp}^{S^{up}}]$, $r_{jq}^{T}=[r_{jq}^{T^{low}},r_{jq}^{T^{up}}]$, $e_{iq}^{T}=[e_{iq}^{T^{low}},e_{iq}^{T^{up}}]$, the distance metrics are derived based on the specific positional relationships between the actual and expected value intervals, as expressed below:


$$d_{jip}^{S}=\{\begin{array}{cc} {}*20c(0.5r_{ip}^{S^{low}}+e_{jp}^{S^{up}})-e_{jp}^{S^{up}} & e_{jp}^{S^{up}}<r_{ip}^{S^{low}}\;\\ (0.5r_{ip}^{S^{low}}+r_{ip}^{S^{up}})-e_{jp}^{S^{low}} & \;r_{ip}^{S^{up}}<e_{jp}^{S^{low}}\\ 0.5(r_{ip}^{S^{up}}-e_{jp}^{S^{up}}) & e_{jp}^{S^{low}}<r_{ip}^{S^{low}}\leq e_{jp}^{S^{low}}<r_{ip}^{S^{up}}\;\\ 0.5(r_{ip}^{S^{low}}-e_{jp}^{S^{low}}) & r_{ip}^{S^{low}}<e_{jp}^{S^{low}}<r_{ip}^{S^{up}}<e_{jp}^{S^{up}}\\ 0 & e_{jp}^{S^{low}}<r_{ip}^{S^{low}}<r_{ip}^{S^{up}}<e_{jp}^{S^{up}}\;\\ \sqrt{0.5({\left(r_{ip}^{S^{up}}-e_{jp}^{S^{up}}\right)}^{2}+{\left(r_{ip}^{S^{low}}-e_{jp}^{S^{low}}\right)}^{2})} & r_{ip}^{S^{low}}<e_{jp}^{S^{low}}<e_{jp}^{S^{up}}<r_{ip}^{S^{up}}\; \end{array}$$ (43)

For Cloud Models $C_{f}^{S},C_{g}^{T}\in C^{L}$, the distance measure between $\left(r_{ip}^{S},e_{jp}^{S}\right)$ and $\left(r_{jq}^{T},e_{jq}^{T}\right)$ is calculated as follows:


$$\begin{array}{c} d_{jip}^{s}=\frac{\text{1}}{\left|X_{\max}\right|+\left|X_{\min}\right|}|\left(1-\frac{{\left(E{n^{*}}_{r_{ip}^{s}}\right)}^{2}+{\left(H{e^{*}}_{r_{ip}^{s}}\right)}^{2}}{{\left(E{n^{*}}_{r_{ip}^{s}}\right)}^{2}+{\left(H{e^{*}}_{r_{ip}^{s}}\right)}^{2}+{\left(E{n^{*}}_{e_{jp}^{s}}\right)}^{2}+{\left(H{e^{*}}_{e_{jp}^{s}}\right)}^{2}}\right)E{x^{*}}_{r_{ip}^{s}}\\ \;\;-\left(1-\frac{{\left(E{n^{*}}_{e_{ip}^{s}}\right)}^{2}+{\left(H{e^{*}}_{e_{ip}^{s}}\right)}^{2}}{{\left(E{n^{*}}_{r_{ip}^{s}}\right)}^{2}+{\left(H{e^{*}}_{r_{ip}^{s}}\right)}^{2}+{\left(E{n^{*}}_{e_{jp}^{s}}\right)}^{2}+{\left(E{n^{*}}_{r_{ip}^{s}}\right)}^{2}}\right) \end{array}$$ (44)

The value functions of robots $T_{j}$ for employees $S_{i}$ on criteria $C_{p}^{S}$ and employees$S_{i}$ for robots $T_{j}$ on criteria $C_{q}^{T}$ can be expressed as follows:


$$V_{jip}^{S}=\{\begin{array}{cc} {}*20c{\left(d_{jip}^{S}\right)}^{\alpha_{1}} & e_{jp}^{S^{up}}<r_{ip}^{S^{low}}\\ -\lambda_{1}{\left(-d_{jip}^{S}\right)}^{\beta_{1}} & r_{ip}^{S^{up}}<e_{jp}^{S^{low}}\\ {\left(0.5(d_{jip}^{S})\right)}^{\alpha_{1}} & e_{jp}^{S^{up}}<r_{ip}^{S^{low}}\leq e_{jp}^{S^{low}}<r_{ip}^{S^{up}}\\ -\lambda_{1}{\left(-d_{jip}^{S}\right)}^{\beta_{1}} & r_{ip}^{S^{low}}<e_{jp}^{S^{low}}\leq r_{ip}^{S^{up}}<e_{jp}^{S^{up}}\\ 0 & e_{jp}^{S^{low}}\leq r_{ip}^{S^{low}}<r_{ip}^{S^{up}}\leq e_{jp}^{S^{up}}\\ {\left(d_{jip}^{S}\right)}^{\alpha_{1}} & r_{ip}^{S^{low}}<e_{jp}^{S^{low}}<r_{ip}^{S^{up}}<e_{jp}^{S^{up}} \end{array}$$ (45)

The estimable parameters α and β govern the concavity and convexity of the value function, with their estimated values from experimental data given by: $\alpha_{1}=\alpha_{2}=\beta_{1}=\beta_{2}=0.88$. Notably, smaller parameter values correspond to stronger risk aversion. The loss aversion parameter λ, where λ>1 indicates greater sensitivity to losses than gains, is conventionally set as: $\lambda_{1}=\lambda_{2}=2.25$ for modeling behavioral preferences.

The aggregate prospect value, representing the aggregating preference of one matching object toward another, is calculated as follows:


$$V_{ji}^{S}=\sum_{p=1}^{f}w_{p}^{S}*v_{jip}^{S}$$ (46)

The criteria weights determined in the previous step play a crucial role in computing the aggregate prospect values.

According to disappointed theory (DT), for a matching pair $\left(S_{i},T_{j}\right)$, if $\exists S_{g}\in S$, $V_{ji}^{S}<V_{jg}^{S}$, robot $T_{j}$ will experience disappointment at not being matched with the higher-utility employee $S_{g}$. Let $D_{ig}^{T_{j}}$ denote the disappointment value between $V_{ji}^{S}$ and $V_{jg}^{S}$. The disappointment value influenced by the sensitivity parameter $\alpha_{j}^{T}$ can be calculated as follows:


$$D_{ig}^{T_{j}}=D_{j}(V_{jg}^{S}-V_{ji}^{S})=1-\exp\{(V_{jg}^{S}-V_{ji}^{S})-In\alpha_{j}^{T}\}$$ (47)

$$D_{ji}^{T}=\sum_{S_{g}\in \Omega_{A}}prob(T_{j},S_{g})D_{ig}^{T_{i}}=\frac{\text{1}}{m}\sum_{S_{g}\in \Omega_{A}}1-\exp\{(V_{jg}^{S}-V_{ji}^{S})-In\alpha_{j}^{T}\}$$ (48)

where $\Omega_{A}=\{S_{g}|V_{ji}^{S}<V_{jg}^{S}\},S_{i},S_{g}\in S,j=1,2,\cdots ,n$. Prior to final matching determination, each robot $T_{j}$ maintains equal matching probability with employee $S_{i}$, expressed as: $prob(T_{j},S_{g})=\frac{\text{1}}{\left|S_{i}\right|}$. The expected value $E_{ji}^{T}$ is then calculated as follows:


$$E_{ig}^{T_{j}}=E_{j}(V_{ji}^{S}-V_{jg}^{S})=\eta_{j}^{T}(1-\exp\{(V_{ji}^{S}-V_{jg}^{S})In\beta_{j}^{T}\})$$ (49)

$$E_{ji}^{T}=\sum_{S_{g}\in \Omega_{B}}prob(T_{j},S_{g})E_{ig}^{T_{j}}=\frac{\text{1}}{m}\sum_{S_{g}\in \Omega_{B}}\eta_{j}^{T}(1-\exp\{(V_{ji}^{S}-V_{jg}^{S})In\beta_{j}^{T}\})$$ (50)

where $\Omega_{B}=\{S_{g}|V_{ji}^{S}>V_{jg}^{S}\}$, $S_{i},S_{g}\in S$,j=1,2,⋯,n. The elation function sensitivity parameter $\beta_{j}^{T}$ governs the elation function: $E_{j}(x)=\eta_{j}^{T}(1-(\alpha_{j}^{T}{)}^{x}),x\geq 0$, where $\eta_{j}^{T}\in \left[0,1\right]$ represents the disappointment avoidance parameter. The parameter ranges are constrained by $0.7\leq \beta_{j}^{T}$ and $\alpha_{j}^{T}\leq 0.9$.

According to the analysis above, the adjusted preference utility for $T_{j}$ is computed as:


$$U_{ji}^{T}=V_{ji}^{S}-D_{ji}^{T}+E_{ji}^{T}$$ (51)

Similarly, the modified preference utility for $S_{i}$ is computed as:


$$U_{ij}^{S}=V_{ij}^{T}-D_{ij}^{S}+E_{ij}^{S}$$ (52)

#### 3.4.2. Preference ranking and satisfaction measurement with individual reference behavior.

Based on the preference utility $U_{ji}^{T}$, the sorted set of potential matching targets for $T_{j}$ is given by $\left\{R_{j1}^{S},R_{j2}^{S},\cdots ,R_{jm}^{S}\right\}$. Similarly, the sorted set for $S_{i}$ is obtained as $\left\{R_{i1}^{T},R_{i2}^{T},\cdots ,R_{in}^{T}\right\}$. Considering the impact of peer effects, individuals typically revise their preference rankings by comparative assessments with similar peers. To describe preference uncertainty, the UPRs are formalized using the relational operators: {{>},{=},{<},{>=<}} with corresponding propositional representations:$\left\{\{H_{1}\},\{H_{2}\},\{H_{3}\},\{H\}\right\}$. Each preference relationship $H_{g}$ is associated with a confidence level $\beta_{g}$, denoted as the pair $\left(H_{g},\beta_{g}\right)$. The complete belief structure takes the form: $\{(H_{1},\beta_{1}),(H_{2},\beta_{2}),(H_{3},\beta_{3}),(H,\beta \}$, which subject to the conditions: $0\leq \beta_{g}\leq 1$, $\sum_{g=1}^{3}\beta_{g}+\beta =1$.

**Fig 5:**
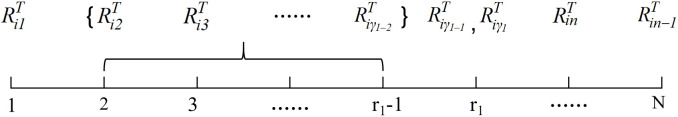
Matching preference ranking of $S_{i}$.


For illustration, when the total comparison count equals n−1 and the UMPRs of $S_{i}$ are shown in Fig 5, the confidence level of each proposition is computed as:

$$R_{i1}^{T}=\{(>,1),(=,0),(<,0),(\Theta ,0)\}$$ (53)

$$R_{i2}^{T}=R_{i3}^{T}=\cdots =R_{i\gamma_{1}-2}^{T}\left\{>,\frac{\left(n-1)-(\gamma_{1}-1\right)}{n-1},\left(=,0\right),\left(<,\frac{\text{1}}{n-1}\right),\left(\Theta ,\frac{(\gamma_{1}-2)-1}{n-1}\right)\right\}$$ (54)

$$R_{i\gamma_{1}-1}^{T}=R_{i\gamma_{1}}^{T}=\left\{\left(>,\frac{n-(\gamma_{1}-1)}{n-1}\right),\left(=,\frac{\text{1}}{n-1}\right),\left(<,\frac{\gamma_{1}-2}{n-1}\right),\left(\Theta ,0\right)\right\}$$ (55)

$$R_{in}^{T}=\left\{\left(>,\frac{n-j}{n-1}\right),\left(=,0\right),\left(<,\frac{j-1}{n-1}\right),\left(\Theta ,0\right)\right\}$$ (56)

The belief structure converted by the adjusted MPRs of $T_{j}$ and $S_{i}$ is represented below:


$$\lambda_{ij}^{T}=\{(H_{1},\beta_{ij}^{T}(H_{1})),(H_{2},\beta_{ij}^{T}(H_{2})),(H_{3},\beta_{ij}^{T}(H_{3})),(H,\beta_{ij}^{T}(H))\}$$ (57)

$$\lambda_{ji}^{S}=\{(H_{1},\beta_{ji}^{S}(H_{1})),(H_{2},\beta_{ji}^{S}(H_{2})),(H_{3},\beta_{ji}^{S}(H_{3})),(H,\beta_{ji}^{S}(H))\}$$ (58)

To calculate the aggregated belief degree of matching objects from both sides, the belief degrees of various propositions can be converted into basic probability assignments according to the evidence reasoning (ER) theory, as illustrated below:


$$\{\begin{array}{c} {}*20cm_{ji}^{T}(Hr)=w_{S}^{T}\cdot \beta_{ji}^{T}(Hr)\\ {\tilde{m}}_{ji}^{T}(H)=w_{S}^{T}\cdot \beta_{ji}^{T}(H)\\ {\bar{m}}_{ji}^{T}(H)=1-w_{S}^{T}\\ m_{ji}^{T}(H)={\bar{m}}_{ji}^{T}(H)+{\tilde{m}}_{ji}^{T}(H) \end{array}$$ (59)

where $w_{S}^{T}$ represents the relative weight assigned by $T_{j}$ to $S_{i}$. Notably, the basic probability of the incompleteness preference relationship H is split into two parts: ${\tilde{m}}_{ji}^{T}(H)$ and ${\bar{m}}_{ji}^{T}(H)$.

The matching satisfaction degree $f_{ij}$ between $S_{i}$ and $T_{j}$ is calculated by combining the belief degrees of aggregated propositions $\beta_{j}^{i}(Hr)$ and $\beta_{j}^{i}(H)$, as shown in the following equations:


$$f_{ij}(\beta_{j}^{i}(H_{1}),\beta_{j}^{i}(H_{2}),\beta_{j}^{i}(H_{3}),\beta_{j}^{i}(H))=\sum_{r=1}^{3}\beta_{j}^{i}(H_{r})\times T(H_{r})+\beta_{j}^{i}(H)\times T(H)$$ (60)

$$\left\{ \begin{array}{c} \beta_{j}^{i}(Hr)=\frac{m_{ij}^{s}⊕m_{ji}^{T}(Hr)}{1-({\bar{m}}_{ij}^{s}⊕{\bar{m}}_{ji}^{T})}\\ \beta_{j}^{i}(H)=\frac{{\tilde{m}}_{ij}^{s}⊕{\tilde{m}}_{ji}^{T}(H)}{1-({\bar{m}}_{ij}^{s}⊕{\bar{m}}_{ji}^{T})} \end{array}\right.$$ (61)

where T(Hr) represents the utility value of the preference relationships. Without loss of generality, we assume $T(H)=T(H_{2})=0.5$, $T(H_{1})=1$, and $T(H_{3})=0$.

### 3.5. Construction and solution of two-stage matching model

For one-to-many bilateral matching, object $T_{j}$ is decomposed into multiple sub-objects ${\hat{T}}_{j}^{\gamma }$, each maintaining identical preference information to enable matching with more $S_{i}$. The decomposed objects can be represented as follows:


$${\hat{T}}_{j}^{\gamma }\in \left\{ \begin{array}{c} T_{1},\;1\leq \gamma \leq \lambda_{1}\\ T_{2},\;\lambda_{1}+1\leq \gamma \leq \lambda_{1}+\lambda_{2}\\ T_{n},\;\lambda_{1}+\lambda_{2}+\cdots +\lambda_{n-1}+1\leq \gamma \leq \lambda_{1}+\cdots \lambda_{n}=c \end{array}\right.$$ (62)

In the initial phase, the primary optimization objectives of the one-to-many BMDM model are to maximize satisfaction degree while minimizing the carbon neutrality costs. To determine the unit carbon dioxide removal cost, we estimate it based on the carbon dioxide removal potential, utilization potential, and profit/loss cost range of ten carbon dioxide removal technologies proposed in [48]. The total carbon dioxide emissions of the production line comprise both direct emissions from employees and indirect emissions form robots. To obtain a more optimal solution, this paper employs a combined satisfaction analysis method [49], which comprehensively considers both the improved matching satisfaction for each matching object and the associated carbon neutrality costs. The equation is presented below:


$$\begin{array}{c} \max Z=\;\sum_{i=1}^{m}\sum_{\gamma =1}^{c}(\delta \left(\frac{f_{i\gamma }}{\text{2}}+\frac{(\frac{G_{i}^{unit(dir)}}{2^{H}}C_{i}^{rem}+G_{\gamma }^{unit(indir)}C_{\gamma }^{rem})t_{i}}{2^{H+1}}\right)\\ +(1-\delta )\sqrt{f_{i\gamma }\cdot \frac{(\frac{G_{i}^{unit(dir)}}{2^{H}}C_{i}^{rem}+G_{\gamma }^{unit(indir)}C_{\gamma }^{rem})t_{i}}{2^{H}}}) \end{array}$$ (M-1)

$$C_{i\gamma }^{rem}=\sum_{r=1}^{R}w_{r}C_{r}^{rem}z_{r}$$ (M-2)

$$w_{r}=\frac{cp_{r}}{\sum {\mathrm{\backslash nolimits}}_{r=1}^{R}cp_{r}z_{r}}$$ (M-3)

$$\sum_{u_{il}^{S}>u_{i\gamma }^{S}}x_{il}+\sum_{u_{g\gamma }^{T}>u_{i\gamma }^{T}}x_{g\gamma }+x_{i\gamma }\leq 1,\;i\in I,\gamma \in J$$ (M-4)

$$\sum_{i=1}^{m}x_{i\gamma }=1,\;i\in I$$ (M-5)

$$\sum_{\gamma =1}^{c}x_{i\gamma }=1,\;\gamma \in J$$ (M-6)

$$x_{i\gamma }\in \{0,1\},\;i\in I,\gamma \in J$$ (M-7)

where δ serves as a mediation parameter that captures the subjective consideration of the decision-makers. The variable $f_{ij}$ represents the satisfaction degree between employee $S_{i}$ and robot $T_{j}$. $G_{i}^{unit(dir)}$ represents the direct carbon dioxide emissions per unit time for employee $S_{i}$, while $C_{i}^{rem}$ denotes the corresponding unit carbon dioxide removal cost for $S_{i}$. Similarly, $G_{\gamma }^{unit(indir)}$ quantifies the indirect carbon dioxide emission per unit times for robot $T_{j}$, with $C_{\gamma }^{rem}$ representing its unit carbon dioxide removal cost. The parameter $t_{i}$ represents the unit assembly time for employee $S_{i}$. The binary decision variable H (taking values 1 or 0) indicates whether $T_{j}$ is matched with multiple $S_{i}$, while N denotes the total number of matched employees. The unit carbon dioxide removal cost $C_{i\gamma }^{rem}$ in models (M-2)–(M-3) depends on the specific technologies employed. For the r−th carbon dioxide removal technology, the cost $C_{r}^{rem}$ applies when the technology is adopted $z_{r}=1$; otherwise, $z_{r}=0$. The importance weight $w_{r}$ reflects each technology’s percentage contribution to the total carbon dioxide removal potential. To ensure stable matching outcomes without blocking pairs, we implement the stability condition (M-4) from reference [50]. Additionally, constraints in (M-5)–(M-7) ensure that each employee is matched with exactly one robot, and each robot is matched with at least one employee. The binary decision variable $x_{i\gamma }$ indicates the matching status between $S_{i}$ and $T_{j}$, where $x_{i\gamma }=1$ if matched and $x_{i\gamma }=0$ otherwise.

**Fig 6:**
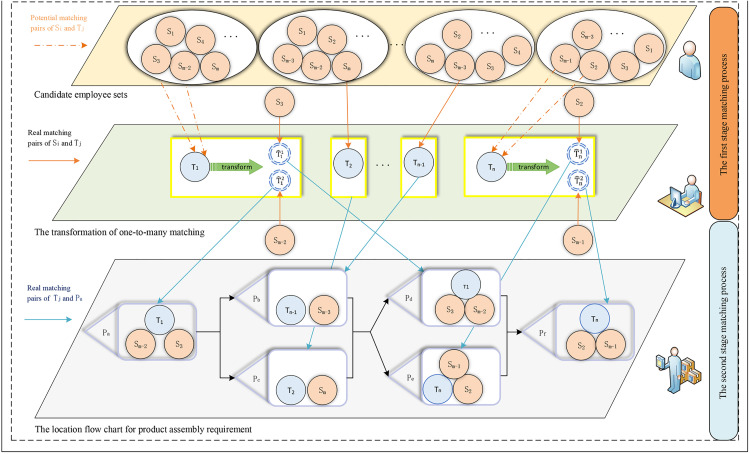
Human-robot collaboration unit allocation for multi-layout workstations.

In the second step, we select the optimal HRCUs for each position by evaluating the correlation between the standardized performance metrics of the position and those of each potential HRCUs. This selection process is designed to create an intelligent, low-carbon production line that minimizes production costs while maintaining high quality and reliability. Since different manufacturing requirements necessitate distinct structural layouts of production lines, including series, parallel, selective, and circular structures, the methodology for calculating comprehensive service attributes varies accordingly. For this study, we utilize the service combination approach depicted in Fig 6.

The appropriate HRCUs for distinct position tasks can be determined using the optimal model illustrated below:


$$\max Z=\sum_{i=1}^{m}\sum_{j=1}^{n}\sum_{u=1}^{n}\left(\psi_{A}Q_{ij}^{u}+\psi_{B}R_{ij}^{u}-\psi_{C}C_{ij}^{u}\right)*x_{ij}$$ (M-8)

$$Q_{ij}^{u}=Q_{il^{\gamma_{p}}}^{a}\cdot Q_{iz^{\gamma_{p}}}^{b}\cdot Q_{il^{\gamma_{q}}}^{c}\cdot (\theta_{k}\cdot Q_{io^{\gamma_{p}}}^{d}+(1-\theta_{k})\cdot Q_{io^{\gamma_{q}}}^{e})\cdot Q_{il^{\gamma_{q}}}^{f}$$ (M-9)

$$R_{ij}^{u}=R_{il^{\gamma_{p}}}^{a}\cdot R_{iz^{\gamma_{p}}}^{b}\cdot R_{il^{\gamma_{q}}}^{c}\cdot (\theta_{k}\cdot R_{io^{\gamma_{p}}}^{d}+(1-\theta_{k})\cdot R_{io^{\gamma_{q}}}^{e})\cdot R_{il^{\gamma_{q}}}^{f}$$ (M-10)

$$C_{ij}^{u}=C_{il^{\gamma_{p}}}^{a}\cdot C_{iz^{\gamma_{p}}}^{b}\cdot C_{il^{\gamma_{q}}}^{c}\cdot (\theta_{k}\cdot C_{io^{\gamma_{p}}}^{d}+(1-\theta_{k})\cdot C_{io^{\gamma_{q}}}^{e})\cdot C_{il^{\gamma_{q}}}^{f}$$ (M-11)

$$Q_{\min}^{quality}\leq Q_{ij}^{u}$$ (M-12)

$$R_{\min}^{quality}\leq R_{ij}^{u}$$ (M-13)

$$C_{ij}^{u}\leq C_{\max}^{\cos t}$$ (M-14)

where $P_{u}\in \{a,b,c,d,e,f\}$ represents the connection positions, including serial, parallel and selective structure, $Q_{il^{\gamma_{p}}}^{a}$ denotes the matching pair between the i−th employee and l−th robot assigned to a−th position for task completion j={l,z,o,l≠z,k≠o}. The virtual objects $l^{\gamma_{p}}$ and $l^{\gamma_{q}}$ are derived from the same robot $T_{l}$.

**Fig 7:**
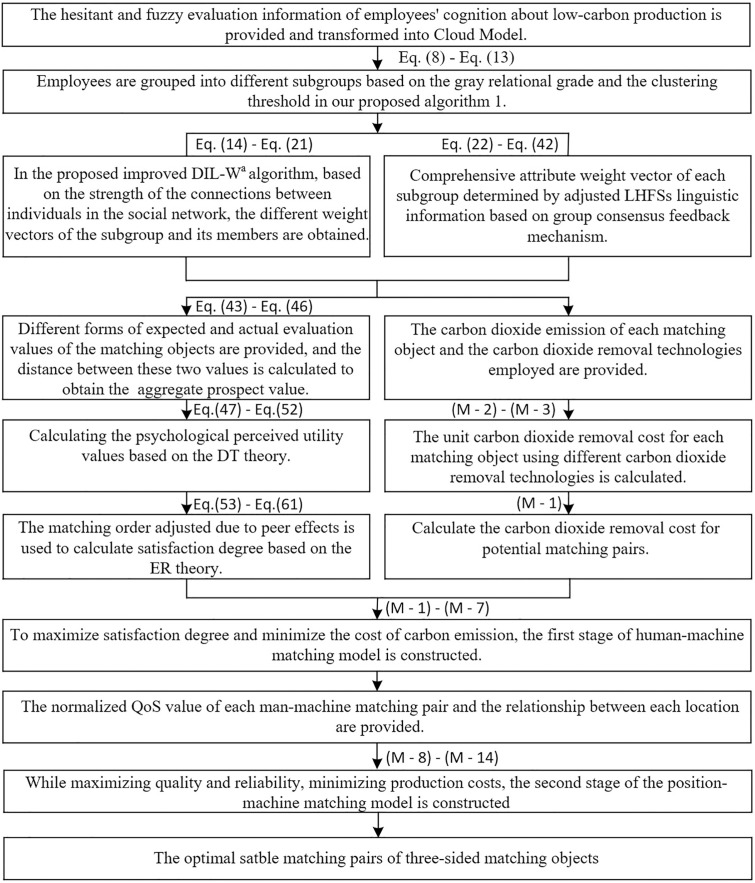
The procedure of the proposed model.

For each matching pair at a−th position, the parameters $Q_{il^{\gamma_{p}}}^{a}$, $R_{il^{\gamma_{p}}}^{a}$ and $C_{il^{\gamma_{p}}}^{a}$ represent the quality level, reliability, and production cost, respectively. The threshold constraints for $Q_{ij}^{u}$, $R_{ij}^{u}$ and $C_{ij}^{u}$ are specified in (M-12)–(M-14). The probability of selecting the k−th service from a sequentially structured service set is denoted by $\theta_{K}$. The mediation parameter $\psi_{A}$, $\psi_{B}$ and $\psi_{C}\in [0,1]$ are assigned to reflect the relative importance of different objectives, subject to the condition $\psi_{A}+\psi_{B}+\psi_{C}=1$. The proposed model is solved using the branch-and-bound algorithm implemented in the Lingo mathematical optimization software. As previously mentioned, the method’s procedure is illustrated in Fig 7.

## 4. Case study

In this illustrate case, an automobile manufacturer has submitted historical performance data and evaluations for eight employees $\left\{S_{1},S_{2},\cdots ,S_{8}\right\}$ and six robots $\left\{T_{1},T_{2},\cdots ,T_{6}\right\}$ to a third-party service platform. The objective is to construct a low-carbon automotive assembly line by optimally matching these resources to six key positions $\left\{U_{1},U_{2},\cdots ,U_{6}\right\}$, each with distinct tasks.

The implementation process consists of several steps: First, six domain experts evaluated employee competencies in low-carbon production using a seven-level linguistic term set detailed in Table A.2 (see S1 File), with evaluation criteria weighted at {0.2,0.35,0.35,0.1}. The CWA operator was then applied to compute aggregated Cloud Models of the evaluation data, with results presented in Table A.3 (see S1 File). An intuitive trust matrix $V=(V_{kf}{)}_{l\times l}$ between experts was also provided in Table A.4 (see S1 File) to determine expert weight $w_{k}^{E}$.

Next, gray relational grades were calculated for each employee $S_{i}$ shown in Table 1. Using Algorithm 1 with parameters φ=0.8 and L=3, employees were clustered until meeting the termination condition $N_{l}\leq 3$. This process yielded three employee subgroups:$\gamma_{1}=\{S_{6},S_{7},S_{8}\}$, $\gamma_{2}=\{S_{2},S_{3},$$S_{5}\}$, and $\gamma_{3}=\{S_{1},S_{4}\}$.

**Table 1:** Gray relational grade and Δgi values.

$S_{g}$	$S_{i}$	$E_{1}$	$E_{1}$	$E_{1}$	$E_{1}$	$E_{1}$	$E_{1}$	The fray relational grade
$S_{1}$	$S_{2}$	0.218	0.218	0.218	0.218	0.218	0.218	0.715
$S_{1}$	$S_{3}$	0.003	0.034	0.121	0.202	0.029	0.031	0.718
$S_{1}$	$S_{4}$	0.016	0.225	0.074	0.099	0.013	0.075	0.744
$S_{1}$	$S_{5}$	0.058	0.026	0.006	0.031	0.066	0.022	0.643
⋮	⋮	…	…	…	…	…	…	…
$S_{7}$	$S_{g}$	0.038	0.067	0.167	0.031	0.233	0.248	0.644

**Fig 8:**
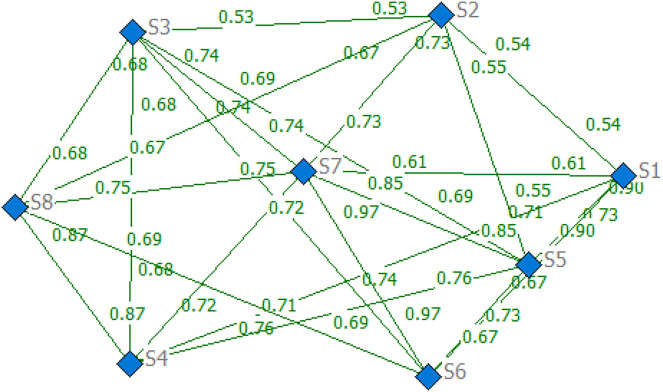
Social network of employee relationships.

**Fig 9:**
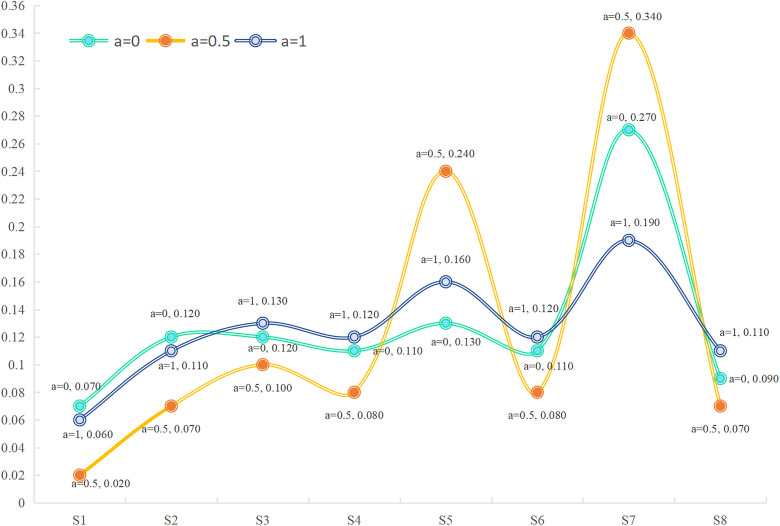
The weights of individuals with different α.

Based on the case data, the social network of employees in Fig 8 is utilized to calculate the weights of the employees across different coefficients α using an enhanced $DIL-w^{\alpha }$ algorithm. The precise results are represented in Table 2 (see S1 File) and Fig 9. Before determining the weights of $C_{q}^{T}$ provided by different subgroup, employees evaluated the importance of indicator $C_{q}^{T}$ using LHFSs, as documented in the Table A.5 (see S1 File). Our proposed improved group consensus feedback adjustment mechanism yields subgroup weights for α=0.5, with the exact weight vector $\left\{w_{\gamma_{1}}=0.490,w_{\gamma_{2}}=0.410,w_{\gamma_{3}}=0.100\right\}$.

**Table 2:** Node centrality and average weight.

Note	$C_{\bar{w}}^{\alpha }(S_{i})$			$C_{D}^{\alpha }(S_{i})$		
	α=0	α=0.5	α=1	α=0	α=0.5	α=1
$S_{1}$	1	0.84	0.70	5	4.18	3.49
$S_{2}$	1	0.78	0.60	5	3.89	3.02
$S_{3}$	1	0.82	0.68	6	4.94	4.06
$S_{4}$	1	0.86	0.75	5	4.32	3.74
$S_{5}$	1	0.86	0.86	6	5.15	4.42
$S_{6}$	1	0.87	0.87	5	4.36	3.80
$S_{7}$	1	0.95	0.95	6	5.68	5.37
$S_{8}$	1	0.86	0.86	5	4.28	3.66

To obtain attribute weights with higher group consensus through this feedback mechanism, we calculate aggregate Cloud Models using the CHA operator for LHFS information on $C_{q}^{T}$ from both subgroups and all individuals, following Eq (8) – (11). These aggregated results appear in Table A.6 (see S1 File). Subsequently, we compute the initial total consensus degree at three levels using Eq (28) – (31).

**Fig 10:**
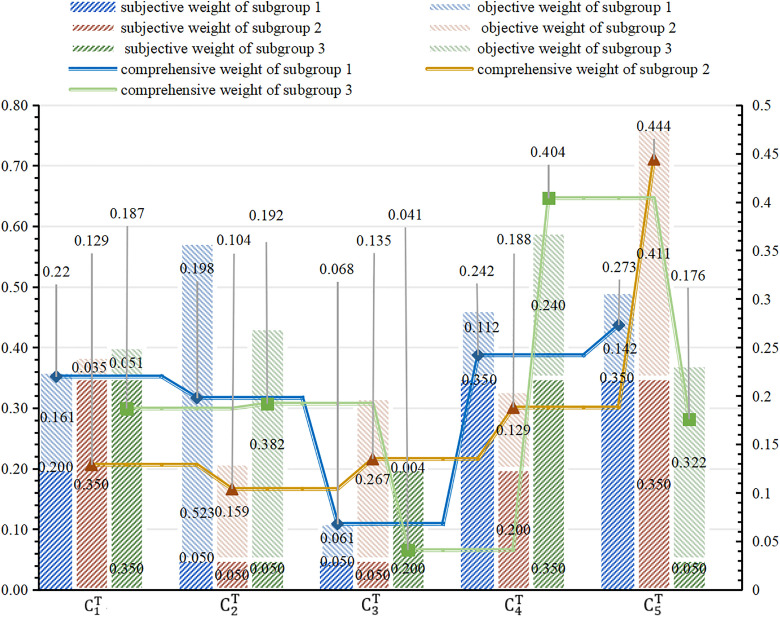
Weights of three types given by different subgroups for $C^{T}$.

As shown in Table 3, value of $CG^{\left(0\right)}=0.734$ falls below the group consensus threshold ε=0.8. Consequently, the corresponding evaluation elements $\left(s_{l}^{\gamma },C_{q}^{T}\right)$ require adjustment to achieve higher group consensus degree, as shown in Table 4. Three distinct group consensus levels for adjusted values are calculated following the process described in Fig 4, with the final evaluated values presented in Table 5. Using Eq (32) – (43) and the data from Table 5, the subjective weight, objective weight and comprehensive weight of $C_{q}^{T}$ assigned by each subgroup are obtained and shown in Fig 10.

**Table 3:** Three-level group consensus degrees (φ=0).

	CE	CS	CG	CG
$\gamma_{1}$	$\left(\begin{array}{ccccc} {}*20c0.750 & 0.806 & 0.801 & 0.760 & 0.775\\ 0.786 & 0.877 & 0.792 & 0.763 & 0.874\\ 0.776 & 0.995 & 0.898 & 0.823 & 0.761 \end{array}\right)$	(0.778,0.818,0.851)	0.816	(1.247,0.737,0.132), (1.291,0.391,0.202), (0.871,0.391,0.202), (1.360,0.455,0.143), (1.595,0.498,0.092).
$\gamma_{2}$	$\left(\begin{array}{ccccc} {}*20c0.733 & 0.605 & 0.723 & 0.762 & 0.775\\ 0.702 & 0.605 & 0.792 & 0.729 & 0.849\\ 0.526 & 0.877 & 0.809 & 0.763 & 0.761 \end{array}\right)$	(0.719,0.735,0.747)	0.734	
$\gamma_{3}$	$\left(\begin{array}{ccccc} {}*20c0.753 & 0.883 & 0.792 & 0.762 & 0.761\\ 0.786 & 0.920 & 0.842 & 0.837 & 0.854 \end{array}\right)$	(0.770,0.848)	0.819	

**Table 4:** Adjusted value of parameters $g_{ik^{\prime }}^{\gamma_{l^{\prime }}}$ and ${\bar{g}}_{k^{\prime }}$.

The initial $\left(s_{i}^{\gamma_{l^{\prime }}},C_{q}^{T}\right)$ and ${\bar{g}}_{k^{\prime }}^{\left(0\right)}$	The initial $\left(s_{i}^{\gamma_{l^{\prime }}},C_{q}^{T}\right)$ and ${\bar{g}}_{k^{\prime }}^{\left(0\right)}$
$g_{51}^{2}=(3.690,1.062,0.271)$	$g_{51}^{2}=(1.519,1.336,0.625)$
$g_{52}^{2}=(3.460,0.840,0.493)$	$g_{52}^{2}=(1.498,1.090,0.814)$
$g_{53}^{2}=(1.384,0.840,0.493)$	$g_{53}^{2}=(1.055,1.090,0.814)$
$g_{31}^{2}=(2.667,1.183,0.150)$	$g_{31}^{2}=(1.375,1.376,0.531)$
$g_{32}^{2}=(3.460,0.840,0.493)$	$g_{32}^{2}=(1.498,1.090,0.814)$
$g_{34}^{2}=(2.317,1.026,0.364)$	$g_{34}^{2}=(1.344,1.193,0.694)$
$g_{21}^{2}=(3.075,1.062,0.271)$	$g_{21}^{2}=(1.435,1.336,0.625)$

**Table 5:** Three-level group consensus degrees (φ=1).

	CE	CS	CG	CG
$\gamma_{1}$	$\left(\begin{array}{ccccc} {}*20c0.798 & 0.751 & 0.772 & 0.911 & 0.775\\ 0.797 & 0.961 & 0.790 & 0.810 & 0.874\\ 0.753 & 0.858 & 0.836 & 0.781 & 0.761 \end{array}\right)$	(0.801,0.847,0.798)	0.815	(0.634,0.817,0.353), (0.744,0.455,0.294), (0.806,0.435,0.267), (1.280,0.468,0.169), (1.595,0.498,0.092).
$\gamma_{2}$	$\left(\begin{array}{ccccc} {}*20c0.805 & 0.883 & 0.876 & 0.788 & 0.775\\ 0.805 & 0.883 & 0.790 & 0.765 & 0.849\\ 0.802 & 0.961 & 0.865 & 0.810 & 0.761 \end{array}\right)$	(0.825,0.818,0.840)	0.828	
$\gamma_{3}$	$\left(\begin{array}{ccccc} {}*20c0.753 & 0.887 & 0.796 & 0.788 & 0.761\\ 0.797 & 0.851 & 0.755 & 0.875 & 0.854 \end{array}\right)$	(0.797,0.827)	0.812	

**Fig 11:**
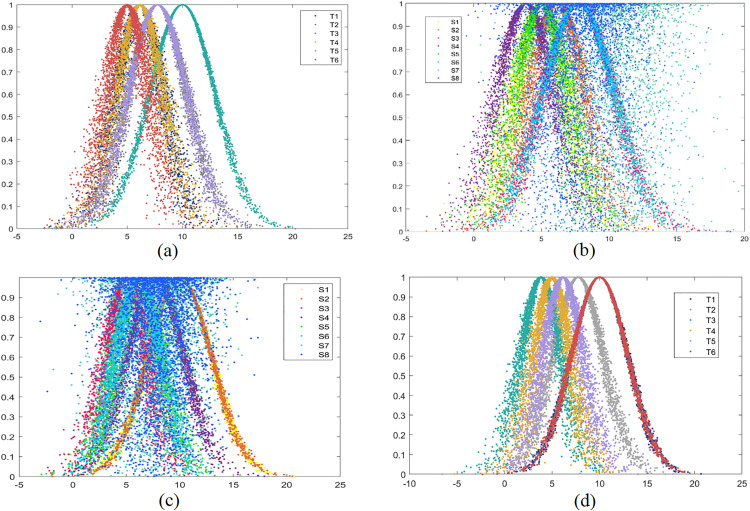
Cloud model representation of expected and actual values in human-robot collaboration. (a): Cloud Expected cooperation ability. (b): Actual cooperation ability. (c): Expected interface operability. (d): Actual interface operability.

The automobile manufacturer has provided the basic expectations and actual values of employees for five criteria to the third-party service platform, as presented in Table A.7 (see S1 File) – Table A.8 (see S1 File) and Fig 11(a)-(b). Similarly, the values of robot agents as provided by employees are shown in Table A.9 (see S1 File) – Table A.10 (see S1 File) and Fig 11 (b)-(d).

The psychological perceived utility values are calculated using Eq (44) – (53), establishing the foundational ranking for matching objects. However, it has been observed that peer effects significantly influence an individual’s decision-making process. Consequently, the model permits each matching object to adjust its MPRs for potential targets according to subgroup member evaluations. The adjusted MPRs for both $S_{i}$ and $T_{j}$ are shown in Tables 6 and 7.

**Table 6:** Adjusted matching preference rankings for employees.

	Adjusted ranking of $S_{i}$		Adjusted ranking of $S_{i}$
$T_{1}$	$\left\{S_{1},S_{4}\}>S_{7}>S_{8}>S_{6}>\{S_{2},S_{3},S_{5}\right\}$	$T_{4}$	$S_{8}>S_{6}>S_{1}>S_{7}>S_{4}>S_{5}>S_{3}>S_{2}$
$T_{2}$	$S_{4}>S_{1}>\{S_{6},S_{7}\}>S_{8}>S_{3}>\{S_{2},S_{5}\}$	$T_{5}$	$S_{6}>S_{8}>S_{4}>S_{7}>S_{1}>>S_{3}>S_{5}>S_{2}$
$T_{3}$	$\{S_{1},S_{4}\}>S_{7}>\{S_{6},S_{8}\}>S_{3}>S_{5}>S_{2}$	$T_{6}$	$S_{8}>S_{6}>S_{7}>\{S_{1},S_{4}\}>S_{5}>S_{3}>S_{2}$

**Table 7:** Adjusted matching preference rankings for robots.

	Adjusted ranking of $S_{i}$		Adjusted ranking of $S_{i}$
$S_{1}$	$T_{6}>T_{3}>\{T_{2},T_{5}\}>T_{4}>T_{1}$	$S_{5}$	$T_{6}>T_{3}>T_{2}>T_{5}>T_{4}>T_{1}$
$S_{2}$	$T_{6}>T_{3}>T_{2}>T_{5}>T_{4}>T_{1}$	$S_{6}$	$T_{6}>T_{3}>T_{5}>T_{2}>T_{4}>T_{1}$
$S_{3}$	$T_{6}>T_{3}>T_{2}>T_{5}>T_{4}>T_{1}$	$S_{7}$	$T_{6}>T_{3}>T_{5}>T_{2}>T_{4}>T_{1}$
$S_{4}$	$T_{6}>T_{3}>\{T_{2},T_{5}\}>T_{4}>T_{1}$	$S_{8}$	$T_{6}>T_{3}>T_{5}>T_{2}>T_{4}>T_{1}$

The total matching satisfaction degree is calculated using the adjusted MPRs of both $S_{i}$ and $T_{j}$ through Eq (54) – (62).

Following the calculation of modified matching satisfaction degree and carbon neutrality costs for each matching pair, with parameters $w_{T}^{S}=0.5$ and ψ=0.5, the matrix $D=[D_{i\gamma }{]}_{8\times 8}$ is calculated as follows:


$$D=\left[\begin{array}{cccccccc} {}*20c3.209 & 1.000 & 1.000 & 2.631 & 3.625 & 1.224 & 1.224 & 3.209\\ 4.727 & 1.910 & 1.910 & 5.819 & 5.819 & 1.977 & 1.977 & 5.819\\ 4.723 & 1.902 & 1.902 & 3.612 & 5.411 & 1.608 & 1.608 & 3.153\\ 5.468 & 1.989 & 1.989 & 4.774 & 6.592 & 2.130 & 2.130 & 5.468\\ 4.554 & 1.830 & 1.830 & 3.825 & 5.019 & 1.691 & 1.691 & 3.005\\ 6.342 & 1.678 & 1.678 & 4.035 & 4.437 & 1.120 & 1.120 & 3.574\\ 6.342 & 1.678 & 1.678 & 3.670 & 5.223 & 1.396 & 1.396 & 3.574\\ 7.889 & 2.640 & 2.640 & 5.336 & 4.830 & 1.766 & 1.766 & 4.830 \end{array}\right]$$

The optimization model was solved by Lingo 11.0 software, which generated an optimal objective function value and produced the final matching results as follows:


$$X^{\prime T}=\left[\begin{array}{cccccccc} {}*20c0 & 0 & 0 & 0 & 0 & 0 & 0 & 1\\ 0 & 0 & 0 & 1 & 0 & 1 & 0 & 0\\ 1 & 0 & 0 & 0 & 0 & 0 & 0 & 0\\ 0 & 0 & 0 & 0 & 0 & 0 & 1 & 0\\ 0 & 0 & 1 & 0 & 1 & 0 & 0 & 0\\ 0 & 1 & 0 & 0 & 0 & 0 & 0 & 0 \end{array}\right]$$

The matching matrix yields the optimal assignment between eight employees and six robots as $\{(S_{8},T_{1}),(S_{1},S_{6},T_{2}),(S_{1},T_{3}),(S_{7},T_{4}),(S_{3},S_{5},T_{5}),(S_{2},T_{6}\}$. This configuration indicates that robots $T_{2}$ and $T_{5}$ are collaboratively operated by two employees respectively to complete the task. The solution achieves a total matching satisfaction degree of 5.103 with a minimal carbon neutrality cost of 153.966.

Furthermore, it is imperative to appropriately match HRCUs and their assigned positions based on the normalized values in Table A.11 (see S1 File) and the operational relationships between different positions, as illustrated in the previous example.

Without loss of generality, we set $\psi_{1}=\psi_{2}=\psi_{3}=\frac{\text{1}}{\text{3}}$. The matrix of matching result presented below was obtained by solving the optimization model (M-8)–(M-14). The optimal objective function value and the corresponding solution from the second matching processes are presented as follows:


$$X^{\prime \prime }=\left[\begin{array}{cccccc} {}*20c0 & 0 & 0 & 0 & 0 & 1\\ 0 & 1 & 0 & 0 & 0 & 0\\ 0 & 0 & 0 & 1 & 0 & 0\\ 0 & 0 & 1 & 0 & 0 & 0\\ 1 & 0 & 0 & 0 & 0 & 0\\ 0 & 0 & 0 & 0 & 0 & 1 \end{array}\right]$$

Based on the assignment matrix $X^{\prime \prime }={\left[x_{ij}^{P}\right]}_{6\times 6}$, the optimal position allocations for HRCUs are determined as: $S_{3},S_{5},T_{5}\to P_{1}$, $S_{4},S_{6},T_{2}\to P_{2}$, $S_{7},T_{4}\to P_{3}$, $S_{1},T_{3}\to P_{4}$, $S_{8},T_{1}\to P_{5}$, $S_{2},T_{6}\to P_{6}$.

## 5. Comparisons and discussions

**Fig 12:**
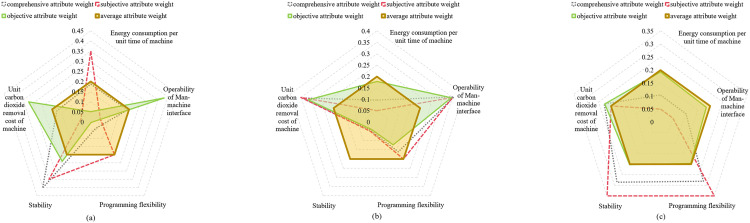
Four types of attribute weights distribution under $\begin{array}{c} \varepsilon =0.7 \end{array}$. (a): Weights by $\gamma_{1}$ subgroup. (b): Weights by $\gamma_{2}$ subgroup. (c): Weights by $\gamma_{3}$ subgroup.

**Fig 13:**
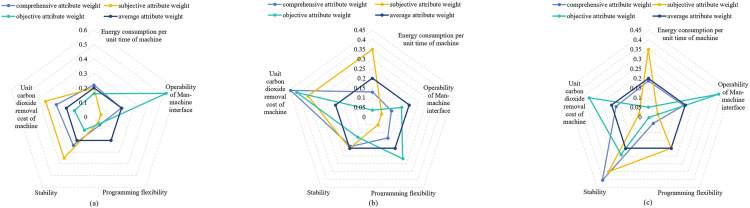
Four types of attribute weights distribution under $\begin{array}{c} \varepsilon =0.8 \end{array}$. (a): Weights by $\gamma_{1}$ subgroup. (b): Weights by $\gamma_{2}$ subgroup. (c): Weights by $\gamma_{3}$ subgroup.

This section presents comparative analyses to validate the superiority and innovation of our proposed model. The experimental parameters are configured as follows:

(i) Group consensus thresholds ε={0.7,0.8}.
(ii) Fixed attribute weights for employees: $w_{P}^{S}=0.2$.
(iii) Four weight vectors for $C^{T}$ were calculated using Eq (32) – (43). The specific values for different consensus thresholds ε={0.7,0.8} are presented in Figs 12–13.

We evaluate against three benchmark models:

Model I: Conventional matching without ER and DT theory, following the approach described in Reference [51].
Model II: The psychological perception-based matching model proposed in [33].
Model III: A matching model that exclusively incorporates peer-effect influence factors from group members while excluding psychological perception components.

Proposed Model: integrates DT theory and ER theory to compute matching satisfaction scores through adjusted UMPRs.

**Fig 14:**
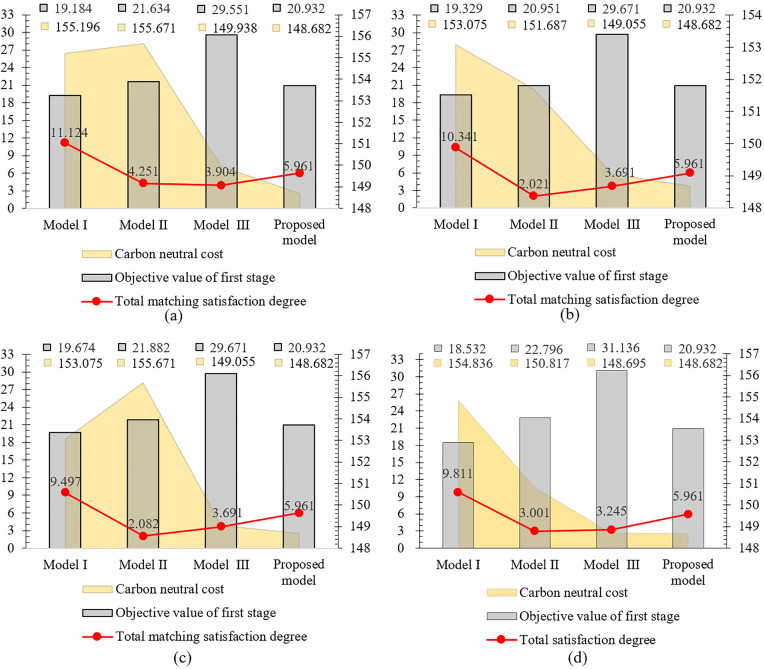
Performance comparison of three indicators across different weighting schemes under $\begin{array}{c} \varepsilon =0.7 \end{array}$. (a): Comprehensive attribute weights $w_{k^{\prime }}^{\gamma_{l^{\prime }}^{*}}$. (b): Subjective attribute weights $(w_{k^{\prime }}^{\gamma_{l^{\prime }}}{)}_{s}$. (c): Objective attribute weights $(w_{k^{\prime }}^{\gamma_{l^{\prime }}}{)}_{o}$. (d): Average attribute weights $(w_{k^{\prime }}^{\gamma_{l^{\prime }}}{)}_{a}$.

**Fig 15:**
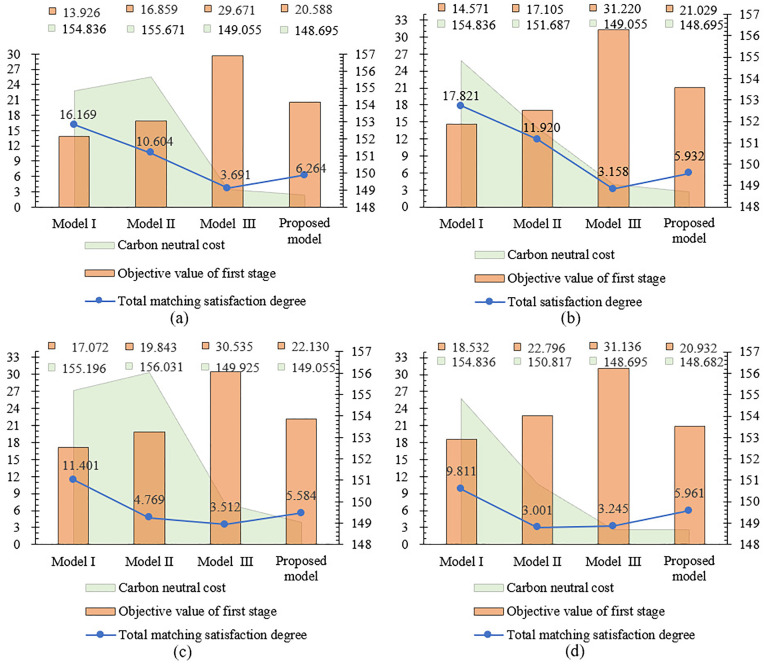
Performance comparison of three indicators across different weighting schemes under $\begin{array}{c} \varepsilon =0.8 \end{array}$. (a): Comprehensive attribute weights $w_{k^{\prime }}^{\gamma_{l^{\prime }}^{*}}$. (b): Subjective attribute weights $(w_{k^{\prime }}^{\gamma_{l^{\prime }}}{)}_{s}$. (c): Objective attribute weights $(w_{k^{\prime }}^{\gamma_{l^{\prime }}}{)}_{o}$. (d): Average attribute weights $(w_{k^{\prime }}^{\gamma_{l^{\prime }}}{)}_{a}$.

The proposed model demonstrates comparative performance against the aforementioned models across three critical indicators: (1) carbon neutrality costs, (2) total satisfaction degree, and (3) first-phase matching objective values. These comparative results are presented in Figs 14–15.

Simulation Experiment Ⅰ: With fixed group consensus threshold ε, we compare three performance indicators across four attribute weight configurations, as presented in Figs 14–15.

Simulation Experiment Ⅱ: Using comprehensive attribute weights of $C^{T}$ and group consensus degree thresholds ε={0.7,0.8} for utility value calculations, the comprehensive results are illustrated in Figs 14(a) and 15(a).

Simulation Experiment Ⅲ: Foe each of the four attribute weights, we analyze matching results between consensus thresholds ε=0.7 shown in Fig 14 and ε=0.8 shown in Fig 15.

**Fig 16:**
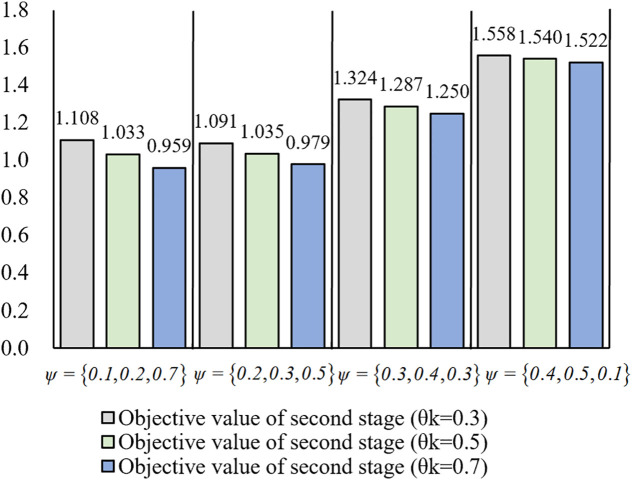
Comparative analysis of second-phase objective values under varied $\psi_{i}$ and $\theta_{k}$.

Simulation Experiment Ⅳ: We define the probability parameter as $\theta_{k}\in \{0.3,0.5,0.7\}$ and mediation parameters as $\psi_{1},\psi_{2},\psi_{3}\in [0,1]$, subject to the constraint $\sum_{i=1}^{3}\psi_{i}=1$. Normalized comparative results across varied $\psi_{i}$ and $\theta_{k}$ combinations are presented in Fig 16.

The experimental results demonstrate significant variations in performance across the evaluated models. As shown in Fig 14(a) and Fig 15(a), Model I demonstrates consistently higher matching satisfaction values compared to Model II and Model III. This phenomenon reveals the limitations of applying either theory in isolation: DT theory’s quantification of negative emotions appears to adversely affect outcomes, while ER theory’s conservative evidence fusion mechanism potentially constrains matching flexibility. Notably, our proposed Model IV, which applies both theories, demonstrates superior performance with a 40.2% improvement in satisfaction over Model II and a 52.6% enhancement compared to Model III. The improved performance of the proposed model can be primarily attributed to the retention of highly credible matching results based on ER theory, while simultaneously ensuring psychological realism by quantifying affective factors using DT theory. Further analysis reveals that although Model I achieved the highest satisfaction, exhibited two critical limitations: (1) significantly higher carbon neutrality costs compared to Model IV, and (2) failure to incorporate psychological perception mechanisms and peer effects. These omissions resulted in a measurable discrepancy between evaluated and actual satisfaction levels, and reduced matching stability. These findings confirm the advantages of the proposed model in balancing satisfaction, environmental costs, and matching reliability.

The group consensus threshold is designated as ε=0.8 in Fig 15(a)-(d). Comparative analysis reveals that all models achieve optimal initial matching target values when employing comprehensive attribute weights, with our proposed model attaining the highest total satisfaction degree through this weighting approach. While the carbon neutrality cost calculated using comprehensive weights in the proposed model is higher than the minimum cost obtained with average attribute weights shown in Fig 15(d), the enhanced satisfaction level demonstrated in Fig 15(a) makes the comprehensive weighting scheme preferable.

Comparative analysis between Figs 14(a) and 15(a) reveals that increasing the group consensus threshold from ε=0.7 to ε=0.8 elevates matching satisfaction from 5.961 to 6.264. This outcome indicates that enhanced consensus requirements during attribute weight evaluation strengthen employees’ cognitive alignment with the assessment outcomes, thereby improving acceptance of matching results and ensuring the final allocations better reflect collective preferences.

Fig 16 reveals two critical parameters in the second-step matching process. First, for a fixed set of ψ, the objective value of second step decreases as the selection probability $\theta_{k}$ increases, indicating that prioritizing matches with $P_{d}$, reduces the objective value of second-step matching. Conversely, emphasizing $P_{e}$ over $P_{d}$, yields superior matching outcomes. Second, when $\theta_{k}$ remains constant, increasing both $\psi_{1}$ and $\psi_{2}$ elevates the objective value of second step, demonstrating that greater emphasis on quality and reliability enhances matching effectiveness.

A detailed comparison between Model I and our proposed model was conducted using the evaluation metrics from Fig 15(a). While Model I demonstrates higher matching satisfaction scores, its matching result: $u^{\prime }=\{(S_{2},T_{1}),(S_{4},S_{7},T_{2}),(S_{8},T_{3}),(S_{3},T_{4}),$$(S_{5},S_{6},T_{5}),(S_{1},T_{6}\}$ contains an unstable blocking pair $\left(T_{6},S_{4}\right)$, as evidenced by the preference utility values $u_{46}^{S}=1.765>u_{43}^{S}=1.160$ and $u_{64}^{T}=0.5>u_{61}^{T}=0.473$. This reveals that $S_{4}$ would prefer pairing with $T_{6}$ over its assigned $T_{3}$, while $T_{6}$ also favors $S_{4}$ over its current match $S_{1}$. In contrast, our proposed model eliminates such instabilities by incorporating the stability constraints defined in (M-4), ensuring that no unstable matching pairs exist in the matching results.

## 6. Conclusion

To enhance manufacturing efficiency for complex products in sustainable production systems, a stable one-to-many human-robot matching model that accounts for the carbon neutrality costs associated with various HRCUs is proposed. Furthermore, in accordance with the QoS requirements of tasks, the optimized HRCUs are recommended to workstations with diverse structural layouts. The experimental results demonstrate that the matching decision-making method considering human behavioral factors exhibits superior applicability, significantly enhances matching efficiency, and contributes to the enrichment and development of the theory of bilateral matching under hesitant fuzzy environments. Compared with previous matching methods, this study has made improvements in the following aspects to enhance the accuracy of the matching results.

In the context of the continuous growth in demand for customized complex products, a stable one-to-many matching model is proposed. The potential carbon emissions resulting from different human-robot matching pairs are incorporated into the model. Based on task requirements, the optimized HRCUs are further recommended to workstations with diverse structural layouts. This approach effectively addresses the dynamic adjustment needs of hybrid automated production lines in sustainable manufacturing environments. Compared to previous matching methods, the contributions of this paper are concluded as follows:

(1) The uncertainty in evaluation information stems from the fuzziness in linguistic term selection and the difficulty in determining membership degrees. By introducing LHFSs into the expression of uncertain evaluation information, this approach enhances decision-making for human-robot position matching in hesitant fuzzy environments.
(2) This study proposes an innovative social network-based DIL-Wα algorithm that quantifies topological features such as relationship strength and connection density in employee social networks. By integrating information entropy theory with group consensus adjustment mechanisms, we develop an approach to obtain subjective and objective attribute weight evaluation vectors. The final comprehensive attribute weights are derived through least squares method, providing a novel solution for weight assessment in complex social network environments.
(3) This study significantly improves utility value calculation methods by incorporating psychological anticipation effects from potential matching pairings, and innovatively proposes a novel UMPRs evaluation approach. By accounting for individuals’ group-reference behaviors, the method permits assigning identical rankings to partially comparable matching objects and represents non-comparable matching objects as collective sets. This improvement effectively addresses the limitations of traditional models in insufficient consideration of psychological factors and handling of uncertain preferences.
(4) Based on the ER theory [34,35], this study proposes a satisfaction measurement method for UMPRs. The proposed method first converts UMPRs into belief structures comprising multiple propositions with associated confidence levels, subsequently transforming them into basic probability assignments (BPAs). By aggregating BPAs from both matching parties under identical propositions, the method enables precise quantification of satisfaction degrees for potential HRCUs.
(5) This study develops a stable one-to-many human-robot matching model. By optimally pairing workers with collaborative robots that support multi-person interaction, the model effectively improves matching satisfaction in HRCUs while reducing system-level carbon neutrality costs. Moreover, stability constraints are incorporated to prevent the formation of blocking pairs among unmatched high-preference agents.

This study still has certain limitations, which are primarily summarized as follows: (1) The robot’s evaluation of human preferences is primarily based on the collaborative performance between personnel and robots in historical tasks, without addressing the scenario in which both parties lack prior cooperation experience; (2) The matched tasks exhibit homogeneity, and the scenario in which certain tasks cannot be executed due to the capability constraints of HRCUs remains unexplored.

Based on the above research, four future directions are identified: (1) The dynamic changes in the number of matching objects on hybrid automated production lines, including the addition of new objects and the removal of weak ones, need to be addressed. (2) The collection of fuzzy and hesitant preference information becomes increasingly challenging with a large number of matching objects. Therefore, intelligent expert evaluation systems must be developed to ensure decision-making efficiency is maintained. (3) In the process of determining the matching priority, the ability of the HRCUs to perform heterogeneous tasks should be fully considered. (4) Non-cooperative behaviors during the consensus adjustment process must be detected and managed promptly to prevent failure in reaching the expected consensus threshold after multiple iterations.

## Supporting information

S1 File Supplementary Tables A.1 to A.11. (DOCX)

## References

[1] Caplan RD. Person-environment fit theory and organizations: Commensurate dimensions, time perspectives, and mechanisms. Journal of Vocational Behavior. 1987;31(3):248–67. doi: 10.1016/0001-8791(87)90042-x

[2] Fan Z-P, Li M-Y, Zhang X. Satisfied two-sided matching: a method considering elation and disappointment of agents. Soft Comput. 2018;22(21):7227–41. doi: 10.1007/s00500-017-2725-1

[3] Yue Q, Ren J, Hu B, Tao Y. Fermatean fuzzy multi-attribute personnel-position matching group decision-making with unknown weight information. Expert Systems with Applications. 2024;237:121451. doi: 10.1016/j.eswa.2023.121451

[4] Yue Q. Bilateral matching decision-making for knowledge innovation management considering matching willingness in an interval intuitionistic fuzzy set environment. Journal of Innovation & Knowledge. 2022;7(3):100209. doi: 10.1016/j.jik.2022.100209

[5] Zhang D, Gong Z, Yan S, Chen Z. Satisfied and fair two-sided matching method considering dual-reference with linguistic preference. Engineering Applications of Artificial Intelligence. 2024;133:108600. doi: 10.1016/j.engappai.2024.108600

[6] Yue Q, Liu L, Tao Y, Huang H. Person-Position Matching Decision Considering Multi-attribute Preferences and Time Factors in Probabilistic Hesitant Fuzzy Environment. Int J Fuzzy Syst. 2025. doi: 10.1007/s40815-024-01939-1

[7] Seenu N, Chetty KRM, Ramya MM, Janardhanan MN. Review on state-of-the-art dynamic task allocation strategies for multiple-robot systems. Ind Robot-Int J Robot Res Appl. 2020;47: 929–42. doi: 10.1108/ir-04-2020-0073

[8] Fathi M, Sepehri A, Ghobakhloo M, Iranmanesh M, Tseng M-L. Balancing assembly lines with industrial and collaborative robots: Current trends and future research directions. Computers & Industrial Engineering. 2024;193:110254. doi: 10.1016/j.cie.2024.110254

[9] Karuppiah K, Sankaranarayanan B, Ali SM, Bhalaji RKA. Decision modeling of the challenges to human–robot collaboration in industrial environment: a real world example of an emerging economy. Flex Serv Manuf J. 2023;35(4):1007–37. doi: 10.1007/s10696-022-09474-7

[10] Tsarouchi P, Makris S, Chryssolouris G. Human–robot interaction review and challenges on task planning and programming. International Journal of Computer Integrated Manufacturing. 2016;29(8):916–31. doi: 10.1080/0951192x.2015.1130251

[11] Acemoglu D, Restrepo P. Automation and New Tasks: How Technology Displaces and Reinstates Labor. Journal of Economic Perspectives. 2019;33(2):3–30. doi: 10.1257/jep.33.2.3

[12] Guo Q, Su Z. The Application of Industrial Robot and the High-Quality Development of Manufacturing Industry: From a Sustainability Perspective. Sustainability. 2023;15(16):12621. doi: 10.3390/su151612621

[13] Itadera S, Domae Y. Motion priority optimization framework towards automated and teleoperated robot cooperation in industrial recovery scenarios. Robotics and Autonomous Systems. 2025;184:104833. doi: 10.1016/j.robot.2024.104833

[14] Dhanda M, Rogers BA, Hall S, Dekoninck E, Dhokia V. Reviewing human-robot collaboration in manufacturing: Opportunities and challenges in the context of industry 5.0. Robotics and Computer-Integrated Manufacturing. 2025;93:102937. doi: 10.1016/j.rcim.2024.102937

[15] Lee E, Barthelmey A, Reckelkamm T, Kang H, Son J. A Study on Human-Robot Collaboration based Hybrid Assembly System for Flexible Manufacturing. In: IECON 2019 - 45th Annual Conference of the IEEE Industrial Electronics Society, 2019. 4197–202. doi: 10.1109/iecon.2019.8927154

[16] Li B, Yang Y, Su J, Liang Z, Wang S. Two-sided matching decision-making model with hesitant fuzzy preference information for configuring cloud manufacturing tasks and resources. J Intell Manuf. 2020;31(8):2033–47. doi: 10.1007/s10845-020-01552-7

[17] Deng Z, Yuan T, Yue Q, Liu X. One-to-many two-sided matching decision of logistics O2O platform considering the intermediary benefit. Knowl Inf Syst. 2025;67(1):689–725. doi: 10.1007/s10115-024-02209-0

[18] Xia M, Xu Z. Hesitant fuzzy information aggregation in decision making. International Journal of Approximate Reasoning. 2011;52(3):395–407. doi: 10.1016/j.ijar.2010.09.002

[19] Torra V. Hesitant fuzzy sets. Int J Intell Syst. 2010;25:529–39. doi: 10.1002/int.20418

[20] Rodriguez RM, Martinez L, Herrera F. Hesitant Fuzzy Linguistic Term Sets for Decision Making. IEEE Trans Fuzzy Syst. 2012;20(1):109–19. doi: 10.1109/tfuzz.2011.2170076

[21] Ertem Z, Veremyev A, Butenko S. Detecting large cohesive subgroups with high clustering coefficients in social networks. Social Networks. 2016;46:1–10. doi: 10.1016/j.socnet.2016.01.001

[22] Backstrom L, Huttenlocher D, Kleinberg J, Lan X. Group formation in large social networks. In: Proceedings of the 12th ACM SIGKDD international conference on Knowledge discovery and data mining, 2006. doi: 10.1145/1150402.1150412

[23] McPherson M, Smith-Lovin L, Cook JM. Birds of a Feather: Homophily in Social Networks. Annu Rev Sociol. 2001;27(1):415–44. doi: 10.1146/annurev.soc.27.1.415

[24] Zhang Z, Kou X, Palomares I, Yu W, Gao J. Stable two-sided matching decision making with incomplete fuzzy preference relations: A disappointment theory based approach. Applied Soft Computing. 2019;84:105730. doi: 10.1016/j.asoc.2019.105730

[25] Arunprasath K, Bathrinath S, Bhalaji RKA, Karuppiah K, Nair A. An integrated approach to modelling of barriers in implementation of cellular manufacturing systems in production industries. Int J Syst Assur Eng Manag. 2023;14(4):1370–8. doi: 10.1007/s13198-023-01941-0

[26] Becker S, Bouzdine-Chameeva T, Jaegler A. The carbon neutrality principle: A case study in the French spirits sector. J Clean Prod. 2020;274:122739. doi: 10.1016/j.jclepro.2020.122739 32834566 PMC7347318

[27] Liu A, Zhu Q, Ji X, Lu H, Tsai S-B, Wang J, et al. Novel Method for Perceiving Key Requirements of Customer Collaboration Low-Carbon Product Design. Int J Environ Res Public Health. 2018;15(7):1446. doi: 10.3390/ijerph15071446 29987252 PMC6069498

[28] Zou G, Li Z, He C. A New Product Configuration Model for Low Product Cost and Carbon-Neutral Expenditure. Sustainability. 2023;15(13):10358. doi: 10.3390/su151310358

[29] Ren M, Ren L, Jain H. Manufacturing service composition model based on synergy effect: A social network analysis approach. Applied Soft Computing. 2018;70:288–300. doi: 10.1016/j.asoc.2018.05.039

[30] Zhang Z, Gao J, Gao Y, Yu W. Two-sided matching decision making with multi-granular hesitant fuzzy linguistic term sets and incomplete criteria weight information. Expert Systems with Applications. 2021;168:114311. doi: 10.1016/j.eswa.2020.114311

[31] Peng H, Zhang H, Wang J, Li L. An uncertain Z-number multicriteria group decision-making method with cloud models. Information Sciences. 2019;501:136–54. doi: 10.1016/j.ins.2019.05.090

[32] Wang P, Xu X, Huang S, Cai C. A Linguistic Large Group Decision Making Method Based on the Cloud Model. IEEE Trans Fuzzy Syst. 2018;26(6):3314–26. doi: 10.1109/tfuzz.2018.2822242

[33] Lu W, Meng Z, Wang Y, Wang Y, Zhai Y. Supply-demand matching in a complex telemedicine environment considering intermediary intervention. Computers & Industrial Engineering. 2022;169:108194. doi: 10.1016/j.cie.2022.108194

[34] Xu Z. An overview of methods for determining OWA weights. Int J Intell Syst. 2005;20(8):843–65. doi: 10.1002/int.20097

[35] Wang J-J, Miao Z-H, Cui F-B, Liu H-C. Robot Evaluation and Selection with Entropy-Based Combination Weighting and Cloud TODIM Approach. Entropy (Basel). 2018;20(5):349. doi: 10.3390/e20050349 33265439 PMC7512868

[36] Wang J, Peng L, Zhang H, Chen X. Method of multi-criteria group decision-making based on cloud aggregation operators with linguistic information. Information Sciences. 2014;274:177–91. doi: 10.1016/j.ins.2014.02.130

[37] Bell DE. Disappointment in Decision Making Under Uncertainty. Operations Research. 1985;33(1):1–27. doi: 10.1287/opre.33.1.1

[38] Loomes G, Sugden R. Disappointment and Dynamic Consistency in Choice under Uncertainty. The Review of Economic Studies. 1986;53(2):271. doi: 10.2307/2297651

[39] Delquié P, Cillo A. Disappointment without prior expectation: a unifying perspective on decision under risk. J Risk Uncertainty. 2006;33(3):197–215. doi: 10.1007/s11166-006-0499-4

[40] Fan Z-P, Zhang X, Chen F-D, Liu Y. Multiple attribute decision making considering aspiration-levels: A method based on prospect theory. Computers & Industrial Engineering. 2013;65(2):341–50. doi: 10.1016/j.cie.2013.02.013

[41] Meng F, Chen X, Zhang Q. Multi-attribute decision analysis under a linguistic hesitant fuzzy environment. Information Sciences. 2014;267:287–305. doi: 10.1016/j.ins.2014.02.012

[42] Liu B, Shen Y, Chen Y, Chen X, Wang Y. A two-layer weight determination method for complex multi-attribute large-group decision-making experts in a linguistic environment. Information Fusion. 2015;23:156–65. doi: 10.1016/j.inffus.2014.05.001

[43] Zhang X, Yang Y, Wang J. Workload balance-based dynamic two-sided matching decision-making approach for cloud manufacturing tasks and services under uncertain preferences. K. 2023;52(11):5087–118. doi: 10.1108/k-03-2022-0306

[44] Opsahl T, Agneessens F, Skvoretz J. Node centrality in weighted networks: Generalizing degree and shortest paths. Social Networks. 2010;32(3):245–51. doi: 10.1016/j.socnet.2010.03.006

[45] Almasi SM, Hu T. Measuring the importance of vertices in the weighted human disease network. PLoS One. 2019;14(3):e0205936. doi: 10.1371/journal.pone.0205936 30901770 PMC6430629

[46] Wu J, Dai L, Chiclana F, Fujita H, Herrera-Viedma E. A minimum adjustment cost feedback mechanism based consensus model for group decision making under social network with distributed linguistic trust. Information Fusion. 2018;41:232–42. doi: 10.1016/j.inffus.2017.09.012

[47] Cheng D, Cheng F, Zhou Z, Wu Y. Reaching a minimum adjustment consensus in social network group decision-making. Information Fusion. 2020;59:30–43. doi: 10.1016/j.inffus.2020.01.004

[48] Hepburn C, Adlen E, Beddington J, Carter EA, Fuss S, Mac Dowell N, et al. The technological and economic prospects for CO2 utilization and removal. Nature. 2019;575(7781):87–97. doi: 10.1038/s41586-019-1681-6 31695213

[49] Zhong-xing W, Shuai H, Fang L. Stable matchings, optimal assignments, and linear programming. Chinese Journal of Mathematics in Practice and Theory. 2014;44:177–83.

[50] Roth AE, Rothblum UG, Vande Vate JH. Stable Matchings, Optimal Assignments, and Linear Programming. Mathematics of OR. 1993;18(4):803–28. doi: 10.1287/moor.18.4.803

[51] Liang Z-C, Yang Y, Liao S-G. Interval-valued intuitionistic fuzzy two-sided matching model considering level of automation. Applied Soft Computing. 2022;116:108252. doi: 10.1016/j.asoc.2021.108252

