# Supplementary material for: A linguistic hesitant fuzzy group decision-making method for sustainable human-robot collaboration
Source: PLoS One. 2025 Oct 9;20(10):e0333758. doi: 10.1371/journal.pone.0333758 (PMC12510669; doi:10.1371/journal.pone.0333758)
Supplement: S1 File — (DOCX) [file pone.0333758.s001.docx]

**Legends for S1 File**

This supporting information file contains all supplementary tables referenced in the main text. Below are the detailed legends for each table.

Legend for Table A.1

Title: Indices, parameters and variables.

Description: This table defines and explains all mathematical notations, parameters, and variables used throughout the study. It provides the conceptual meaning of each item, including sets (e.g., the set of employees or robots), key variables (e.g., weights, consensus levels), and model-specific metrics (e.g., prospect values), within the multi-agent matching decision-making framework.

Legend for Table A.2

Title: Historical evaluation information on employees' cognition of low-carbon production given by experts.

Description: Experts' historical ratings on employees' understanding and awareness of low-carbon production practices. The table is structured by employee and expert, with cell values representing linguistic evaluations.

Legend for Table A.3

Title: The aggregated Cloud Model of historical evaluation information to employees given by experts.

Description: Presents the aggregated cloud model parameters derived from the historical evaluation data in Table A.2. These numerical outputs (e.g., Expectation, Entropy, Hyperentropy) quantitatively represent the evaluation for each employee based on expert inputs.

Legend for Table A.4

Title: The matrix of intuitive trust information between experts $V=(V_{kf})_{l\times l}$.

Description: A matrix displaying the pairwise trust relationships among experts. Each off-diagonal entry represents the degree of intuitive trust one expert has for another.

Legend for Table A.5

Title: The LHFSs information given by individuals about the importance of $C_{q}^{T}$.

Description: Shows the Linguistic Hesitant Fuzzy Sets (LHFSs) provided by individual employees regarding the importance weights of different attributes of robots (Energy consumption per unit time of robot, Interface operability, Programming flexibility, Stability, Unit carbon dioxide removal cost of robot). Each entry contains a set of possible linguistic terms with their associated probability.

Legend for Table A.6

Title: The aggregate Cloud Model of LHFSs information on $C^{T}$ given by subgroups and total individuals.

Description: Provides the aggregated cloud model parameters for attribute importance, derived from the LHFSs in Table A.5. The aggregation is performed at both the subgroup level and the overall group level.

Legend for Table A.7

Title: The expected values of robot agents to employees.

Description: Lists the performance values that robot agents expect from employees across key attributes, including carbon dioxide emission, security coefficient, unit assembly time, and unit carbon dioxide removal cost.

Legend for Table A.8

Title: The self-evaluated values of employees.

Description: Contains self-assessed performance data from employees on the same attributes used in Table A.7, allowing for comparison between employee self-perception and robot expectations.

Legend for Table A.9

Title: The basic expectations to robots given by employees.

Description: Lists the performance values that employees expect from robot agents across key attributes, including energy consumption, programming flexibility, stability, and unit carbon dioxide removal cost.

Legend for Table A.10

Title: Data from self-evaluation of robot agents.

Description: Contains self-assessed performance data from robot agents on the same attributes used in Table A.9, allowing for comparison between robot self-perception and employees expectations.

Legend for Table A.11

Title: Service, quality and reliability value of each position-robot pair.

Description: A comprehensive matrix showing the production cost, qualification rate (as a percentage), and reliability metric (as a percentage) for each possible matching pair between positions and human-robot pairs.
